# Combined MEK and ERK inhibition overcomes therapy-mediated pathway reactivation in *RAS* mutant tumors

**DOI:** 10.1371/journal.pone.0185862

**Published:** 2017-10-05

**Authors:** Mark Merchant, John Moffat, Gabriele Schaefer, Jocelyn Chan, Xi Wang, Christine Orr, Jason Cheng, Thomas Hunsaker, Lily Shao, Stephanie J. Wang, Marie-Claire Wagle, Eva Lin, Peter M. Haverty, Sheerin Shahidi-Latham, Hai Ngu, Margaret Solon, Jeffrey Eastham-Anderson, Hartmut Koeppen, Shih-Min A. Huang, Jacob Schwarz, Marcia Belvin, Daniel Kirouac, Melissa R. Junttila

**Affiliations:** 1 Department of Translational Oncology, Genentech, Inc., South San Francisco, California, United States of America; 2 Department of Biochemical and Cellular Pharmacology, Genentech, Inc., South San Francisco, California, United States of America; 3 Department of Biological Engineering, The Massachusetts Institute of Technology, Cambridge, Massachusetts, United States of America; 4 Department of Oncology Biomarker Development, Genentech, Inc., South San Francisco, California, United States of America; 5 Department of Discovery Oncology, Genentech, Inc., South San Francisco, California, United States of America; 6 Department of Bioinformatics, Genentech, Inc., South San Francisco, California, United States of America; 7 Department of Drug Metabolism and Pharmacokinetics, Genentech, Inc., South San Francisco, California, United States of America; 8 Department of Pathology, Genentech, Inc., South San Francisco, California, United States of America; 9 Department of Discovery Chemistry, Genentech, Inc., South San Francisco, California, United States of America; 10 Department of Cancer Immunology, Genentech, Inc., South San Francisco, California, United States of America; 11 Department of Pre-clinical & Translational Pharmacokinetics Genentech, Inc., South San Francisco, California, United States of America; University of Queensland Diamantina Institute, AUSTRALIA

## Abstract

Mitogen-activated protein kinase (MAPK) pathway dysregulation is implicated in >30% of all cancers, rationalizing the development of RAF, MEK and ERK inhibitors. While BRAF and MEK inhibitors improve *BRAF* mutant melanoma patient outcomes, these inhibitors had limited success in other MAPK dysregulated tumors, with insufficient pathway suppression and likely pathway reactivation. In this study we show that inhibition of either MEK or ERK alone only transiently inhibits the MAPK pathway due to feedback reactivation. Simultaneous targeting of both MEK and ERK nodes results in deeper and more durable suppression of MAPK signaling that is not achievable with any dose of single agent, in tumors where feedback reactivation occurs. Strikingly, combined MEK and ERK inhibition is synergistic in *RAS* mutant models but only additive in *BRAF* mutant models where the RAF complex is dissociated from RAS and thus feedback productivity is disabled. We discovered that pathway reactivation in *RAS* mutant models occurs at the level of CRAF with combination treatment resulting in a markedly more active pool of CRAF. However, distinct from single node targeting, combining MEK and ERK inhibitor treatment effectively blocks the downstream signaling as assessed by transcriptional signatures and phospho-p90RSK. Importantly, these data reveal that MAPK pathway inhibitors whose activity is attenuated due to feedback reactivation can be rescued with sufficient inhibition by using a combination of MEK and ERK inhibitors. The MEK and ERK combination significantly suppresses MAPK pathway output and tumor growth *in vivo* to a greater extent than the maximum tolerated doses of single agents, and results in improved anti-tumor activity in multiple xenografts as well as in two *Kras* mutant genetically engineered mouse (GEM) models. Collectively, these data demonstrate that combined MEK and ERK inhibition is functionally unique, yielding greater than additive anti-tumor effects and elucidates a highly effective combination strategy in MAPK-dependent cancer, such as *KRAS* mutant tumors.

## Introduction

Oncogenic activation of the RAS-RAF-MEK-ERK (MAPK) pathway through overactive growth factor signaling or oncogenic mutation within the *RAS* or *RAF* oncogenes is a central feature in a large number of cancers [1–3]. Lessons from non-oncogenic MAPK signaling illustrates that this pathway is highly regulated to limit and focus signaling. During normal growth factor signaling, distinct pulses of ERK activity lead to translational and transcription events that impact cell morphology and proliferation [4,5]. MAPK signaling is finely tuned to ensure that signal input is tightly correlated with the duration of ERK activation that in turn dictates the commitment of cells to undergo cellular growth and division. Multiple lines of evidence point to both positive and negative feedback mechanisms playing key roles in determining baseline sensitivity to input and maintaining cellular homeostasis [6–11].

In contrast, MAPK-dysregulated tumors are typified by chronically elevated pathway activity resulting in higher basal ERK enzymatic activity, persistent stimulation of transcriptional and translational output, and aberrant cell growth [3]. Targeting of MAPK-dysregulated tumors first showed therapeutic promise in *BRAF*^*V600*^-mutant metastatic melanomas with BRAF inhibitors [12,13]. However, the duration of response of metastatic melanomas to single-agent BRAF inhibition is limited, largely due to MAPK-pathway reactivation, achieved through multiple means [14]. Combinations of BRAF and MEK inhibitors have therefore been used to induce deeper suppression of MAPK signaling in the *BRAF* mutant disease setting to preempt MAPK-pathway reactivation and tumor escape. This strategy has been clinically validated with several combinations of BRAF and MEK inhibitors demonstrating meaningful improvements in progression-free survival and patient outcomes in the *BRAF*^*V600*^-mutant metastatic melanoma setting [15–17].

Unfortunately, *RAS* mutant tumors have not shown the same level of sensitivity to MAPK pathway inhibitors [18–24]. Since BRAF inhibitors are contraindicated in the *RAS* mutant setting due to ‘paradoxical activation’ of signaling [25,26], clinical data has been limited to single agent trials with MEK inhibitors. *RAS* mutant tumors are more susceptible to feedback-mediated pathway reactivation relative to BRAF mutant tumors, evidenced by the observation that single-agent MEK inhibitor treatment results in shallower and more transient suppression of pathway output [22,23]. A kinome shRNA screen identified *CRAF*, *BRAF* and *ERK2* as key determinants of sensitivity to MEK inhibitor [27]. Additionally, feedback-mediated reactivation of the MAPK pathway at the level of ERK has been demonstrated to limit responsiveness to MEK inhibition in the *NRAS* and *KRAS* mutant setting [27–29]. Together these findings indicate that in the context of MEK inhibition, ERK remains a critical node in mediating pathway reactivation and raises the hypothesis that by targeting both nodes concurrently, deeper and more durable efficacy could be attained.

Here we explore the combination of MEK inhibitors with ERK inhibitors as a means to drive deeper and more durable pathway suppression. To this end, we evaluated GDC-0994, a potent and selective small molecule inhibitor of ERK1/2, currently being tested in PhI clinical studies. Like MEK inhibitors, GDC-0994 has broad activity in numerous cell lines and tumor settings, however we show that ERK inhibition is likewise limited by pathway reactivation. We therefore explored the concept of dual node suppression of MEK and ERK based upon the rationalization that this combination would drive deeper and more durable inhibition of MAPK signaling that would delay pathway reactivation enabling increased suppression of cell proliferation and tumor cell death.

## Materials and methods

### Cellular assays

Cobimetinib, GDC-0994 and GDC-0623 were synthesized at Genentech as previously described [30,31]. Other compounds utilized in studies, including VX-11e, SCH772984, and ulixertinib (BVD523), trametinib, selumetinib, and binimetinib were purchased from Selleck Chemicals.

Cell viability studies were performed as previously described [22,30,32]. Cell lines were obtained from the Genentech cell line repository and maintained in RPMI 1640 medium supplemented with 10% FBS and 2 mM L-glutamine. Solutions of each reagent were prepared as 10 mM DMSO stock solutions. For cell viability assays, cells were plated in normal growth medium at 1000–2000 cells per well in 384-well clear-bottom black plates. The following day, compounds were serially diluted 1:2 starting at indicated concentrations, then added to cells in quadruplicates. 96 hours following compound addition, Promega’s CellTiter-Glo^®^ Luminescent Cell Viability reagent was added per manufacturer’s protocol. The NSCLC panel data was generated at ChemPartner (Shanghai, China). For cell death assays, cells were plated in normal growth medium at 2500–3000 cells per well in 96-well clear-bottom black plates. The following day, compounds were added in triplicates at indicated concentrations based on CellTiter-Glo^®^ results. 48 and 72 hours following compound addition, Roche’s Cell Death Detection ELISA^PLUS^ was performed according to manufacturer’s protocol.

For synergy analysis, cells were seeded in 384-well imaging plates (Perkin Elmer, Waltham, MA) and treated with combinations of compounds as described previously [32]. Briefly, an Echo acoustic dispenser (LabCyte Inc, Sunnyvale, CA) was used to transfer each compound at 8 concentrations from 50 μM in decreasing 3-fold dilutions to create all combinations of concentrations with n = 2. Approximately 48 hours after compound addition, 5 μl of 200 μM 5-ethynyl-2'-deoxyuridine (EdU) was added to all wells. After 30 minutes cells were fixed and labeled with Alexa Fluor 647 azide (Life Technologies, Madison, WI) according to the manufacturers instructions and stained with 5 μg/ml Hoechst 33342. EdU-labeled cells were quantitated using an Opera^™^ high-content imaging system and Columbus^™^ image analysis package (Perkin Elmer, Waltham, MA).

Data for percentage of EdU-positive cells was analyzed using Genedata Screener^™^ (Genedata AG, Basel, Switzerland). The predicted additive response for each matrix was calculated according to the Loewe additivity model implemented in Genedata Screener Compound Synergy Extension. Synergy scores were determined as a weighted sum of the values in excess of the predicted additive effects.

### Drug response screening

All cell lines were maintained in RPMI-1640, 5% FBS (10% heat inactivated FBS for suspension lines), and 2 mM glutamine. Cells were plated at a previously determined, line-specific optimal seeding density intended to achieve 75% confluency at 96 hours. After 72 hours compound treatment, viability was determined by Cell Titer-Glo^®^ assay (Promega) according to manufacturer’s instructions. GI_50_ values were determined using a 4-parameter logistic fit with robust outlier detection (Genedata Screener), allowing the lower asymptote to vary between 50 and 100% effect. Three to four independent biological replicates were produced. Same-plate technical replication was observed to have negligible effect on outcome and therefore omitted.

### Tumor protein and RNA isolation

GEM model tumor samples were collected and stored in RNALater (Qiagen, Valencia, CA). Total RNA was extracted with RNeasy Plus Mini kit (Qiagen) following manufacturer’s instructions. RNA quantity was determined using a Nanodrop instrument (Thermo Scientific, Waltham, MA).

### RT-PCR analysis

Transcriptional readouts were assessed using either Fluidigm or Nanostring instruments according to manufacturer’s recommendations. RNA (100 ng) was subjected to cDNA synthesis/preamplification reactions using the Applied Biosystems High Capacity cDNA RT Kit and TaqMan PreAmp Master Mix as per the manufacturer’s protocol (Life Technologies, Carlsbad, CA). Following amplification, samples were diluted one to four with TE and qPCR was conducted on Fluidigm 96.96 Dynamic Arrays using the BioMarkTM HD system according to the manufacturer’s protocol. Cycle threshold (Ct) values were converted to fold change in relative expression values (2^-(ddCt)) by subtracting the mean of the three reference genes from the mean of each target gene followed by subtracting the mean vehicle dCt from the mean sample dCt.

### Immunoblotting

To prepare protein lysates, cells were washed once with ice-cold PBS and lysed in 1X Cell Extraction Buffer (Invitrogen) supplemented with protease inhibitor tablet (Roche Diagnostics, Indianapolis IN) and phosphatase inhibitors (Sigma-Aldrich, St. Louis, MO). Protein concentration was determined using BCA Protein Assay (Thermo Fisher Scientific, Waltham MA). Equal amounts of proteins were resolved by 10% Bis-Tris gels in 1X MOPS running buffer (Invitrogen) and transferred to nitrocellulose membranes (Thermo Fisher Scientific, Waltham MA). Antibodies directed against the following proteins were used: p-ERK, ERK1/2, p-MEK, MEK, cyclinD1, p27, cleaved PARP (Cell Signaling, Danvers MA), p-p90RSK (Abcam, Cambridge MA), GAPDH (Millipore-Sigma, Billerica MA), p90RSK (Thermo Fisher Scientific, Waltham MA). Antigen-antibody interaction was detected with HRP-conjugated goat anti-rabbit and goat anti-mouse antibodies (Jackson ImmunoResearch, Westgrove, PA) using enhanced chemiluminescence detection reagents (Thermo Fisher Scientific, Waltham MA) or with IRDye 800 conjugated, affinity purified anti rabbit IgG (LI-COR Biosciences, Lincoln, NE) and Alexa Fluor 680 goat anti mouse IgG (Thermo Fisher Scientific, Waltham MA) secondary antibodies using a Odyssey Infrared Imaging System (LI-COR Biosciences, Lincoln, NE).

### CRAF knockdown

HCT116 cells were transfected using Amaxa Nucleofector Kit V (Lonza, Basel Switzerland) and following reagents: NTC = non-targeting control oligo #4 (cat.# D-00110-04) CRAF = oligo #3 (cat.# D-003601-03) (Dharmacon, Lafayette CO). 2,000,000 cells were transfected with 2 uM siRNA using Program D-032. Immediately following transfection, cells were plated at 950,000 cells per well in a 4-well plate. 24 hours past electroporation cells were treated with DMSO, 0.25 μM cobimetinib, or 1.25 μM GDC-0994 for 6, 24, or 72 hours.

### Immunoprecipitation-kinase assay

Cells were lysed in NP-40 lysis buffer supplemented with protease inhibitor tablet (Roche Diagnostics, Indianapolis IN) and phosphatase inhibitors (Sigma-Aldrich, St. Louis, MO). Protein concentration was determined using BCA Protein Assay (Thermo Fisher Scientific, Waltham MA). Equal amounts of cell lysates were immunoprecipitated with anti-CRAF (Millipore-Sigma, Billerica MA) and then incubated with 0.4 μg inactive MEK1 (Millipore-Sigma, Billerica MA) in 40 μl of kinase buffer (20 mM MOPS, pH 7.2, 25 mM β-glycerol phosphate, 5 mM EGTA, 1 mM sodium orthovanadate, 1 mM dithiothreitol (DTT), 125 μM ATP, 18 mM MgCl_2_) for 30 min at 30°C with gentle shaking. MEK phosphorylation was detected by immunoblotting with phospho-MEK1/2 antibody (Cell Signaling, Danvers, MA). Quantification was performed using ImageJ.

### Histological analyses

Immunohistochemistry (IHC) was performed on 4 μm thick formalin-fixed paraffin embedded tissue sections mounted on glass slides. All IHC steps were carried out on the Ventana Discovery XT (Ventana Medical Systems; Tucson, AZ) autostainer. For p-p90RSK1 and Ki-67, pre-treatment was done with Cell Conditioner 1, standard time. Primary antibodies Ki-67 (Thermo Fisher Scientific; Waltham, MA) and p-p90RSK1 (Millipore; Billerica, MA) were used at 1:200 and 0.5 ug/mL, respectively. Ki-67 and p-p90RSK1 stained slides were then incubated on slides for 32 and 60 minutes at 37°C, respectively. For cyclin D1 analysis, pre-treatment was done with Cell Conditioner 1, mild time. Primary antibody cyclin D1 (Dako; Carpinteria, CA) was used at the concentration of 0.331 ug/mL and was incubated on slides for 60 minutes at 37°C. Primary cleaved caspase 3 antibody (Cell Signaling; Danvers, MA) was used at a concentration of 0.06 ug/mL and was incubated on slides for 3 hours at room temperature. A biotinylated goat anti-rabbit secondary antibody (Vector; Burlingame, CA) was used at 7.5 ug/mL for 32 minutes. Ventana Rabbit OmniMap (Ventana Medical Systems; AZ) was used as the detection system. Ventana DAB and Hematoxylin II were used for chromogenic detection and counterstain. Whole slide images were acquired with a Nanozoomer 2.0-HT automated slide-scanning platform (Hamamatsu, Hamamatsu City, Shizuoka Pref., Japan) at 200x final magnification. Scanned slides were analyzed in the Matlab software package (version R2014b by Mathworks, Natick, MA) as 24-bit RGB images. Tumor regions were identified using intensity thresholding and morphological filtering. Within tumor regions individual cells were identified using an algorithm based on radial symmetry [33]. Each cell was then scored as positive or negative for DAB staining using a blue-normalized algorithm to identify brown pixels [34].

### In vivo models

The establishment and monitoring of all tumor xenografts and GEM tumor models were performed as previously shown and is described in the full methods section [30,35–37].

### Test material

GDC-0994 was prepared at Genentech as a solution at various concentrations (expressed as free-base equivalents) in 40% PEG400 (polyethylene glycol 400)/60% [10% HP-β-CD (hydroxypropyl-beta-cyclodextrin)]. The vehicle control was 40% PEG400/60% (10% HP-β-CD) or MCT. GDC-0973 was prepared at Genentech as a suspension at various concentrations in methyl-cellulose tween (MCT). GDC-0994, GDC-0973, and vehicle control dosing solutions were prepared once a week for three weeks. The formulations were mixed well by vortexing before dosing. Test articles were stored in a refrigerator set to maintain a temperature range of 4°C–7°C.

### Animal studies

All individuals participating in animal care and use are required to undergo training by the institution's veterinary staff. Any procedures, including handling, dosing, and sample collection mandates training and validation of proficiency under the direction of the veterinary staff prior to performing procedures in experimental in-vivo studies. All animals were dosed and monitored according to guidelines from the Institutional Animal Care and Use Committee (IACUC) on study protocols approved by Genentech’s Laboratory Animal Resource Committee at Genentech, Inc.

#### Drug tolerability studies

To determine the maximum tolerated dose (MTD) of the combination of cobimetinib and GDC-0994, female NCR nude mice (6–8 weeks old) obtained from Taconic (Cambridge City, IN) were administered a various doses of cobimetinib, GDC-0994 or the combination for at least 2 weeks. Drug tolerability was determined by assessing animal condition and body weights. In all in vivo studies, drug treatment was considered intolerable if animals showed signs of morbidity (in consultation with the veterinary staff), including hunching, ruffled fur (where applicable), labored breathing, low body temperature, lack of mobility and/or >20% body weight loss from the time of study start. In animals showing intolerability dosing was halted and animals were monitored for recovery from symptoms and/or were euthanized. This and subsequent in vivo studies identified a recommended combination MTD of cobimetinib at 5 mg/kg and GDC-0994 at 60 mg/kg, however other drug levels and schedules were also found to be tolerable (S7 Fig).

#### Subcutaneous tumor models

All xenograft studies were done as previously described [30]. Briefly, cell lines used for human xenograft studies included HCT116, A549, NCI-H2122, A375.X1, and IPC-298. Cells were grown in normal growth media (RPMI 1640 with L-glutamine and 10% FCS), harvested and implanted subcutaneously into the right flank of female NCR nude mice (6–8 weeks old) obtained from Taconic (Cambridge City, IN) weighing an average of 24–26 g. The mice were housed at Genentech in standard rodent micro-isolator cages and were acclimated to study conditions at least 3 days before tumor cell implantation. Only animals that appeared to be healthy, free of obvious abnormalities and harbored tumors without signs of ulceration were used for each study. Tumor volumes were determined using digital calipers (Fred V. Fowler Company, Inc.) using the formula (L x W x W)/2. Tumor growth inhibition (%TGI) was calculated as the percentage of the area under the fitted curve (AUC) for the respective dose group per day in relation to the vehicle, such that %TGI = 100 x [1 - (AUC_treatment_/day)/(AUC_vehicle_/day)]. Curve fitting was applied to Log_2_ transformed individual tumor volume data using a linear mixed-effects model using the R package nlme, version 3.1–97 in R v2.12.0. Mice were weighed twice a week using a standard scale and checked daily for signs of morbidity as detailed above. Animals were euthanized within 4 hours if deemed moribund or if tumor volumes exceeded 1500mm^3^. Two drug TGI contour analysis (S7a Fig) was performed in R using auto-determined cubic spines with 2 knots, smoothness = 84.

#### Genetically engineered mouse models

We obtained mice from the following institutions: *Kras*^*LSL–G12D*^ from Tyler Jacks (Massachusetts Institute of Technology), *p16/p19*^*fl/fl*^ from Anton Berns (NKI, The Netherlands), *p53*^*frt/frt*^ (Exelixis, Inc.) and *Pdx1-Cre* from Andy Lowy (University of Ohio). All animals are maintained as C57Bl6 strain. Equal numbers of male and female animals were used for experimental cohorts, dosing commenced following confirmation of tumor burden via either ultrasound imaging for PDAC or microCT for NSCLC model and based on baseline tumor volumes animals were equally distributed to treatment arms. All chosen dosing regimens were well tolerated in the GEMMs. Noninvasive imaging and assessment of overall survival were performed as previously described [37]. Animals were monitored daily while on treatment and weights were measured at least twice weekly. Progression free survival (PFS) was determined based on the time of tumor size doubling or death. Date of death was based either on mortality or pre-determined morbidity criteria for euthanasia, as detailed above. If deemed moribund, animals were euthanized within 1–4 hrs. In PFS plots, 8 out of 42 animals were found dead in PDAC and 10 out of 59 in NSCLC. Treatment of mice was continuous until all animals were terminated. Cobimetinib and GDC-0994 were dosed at 5 mg/kg and 60 mg/kg by oral gavage (PO), daily (QD) for the PDAC and NSCLC GEMM studies.

### Statistical analyses

Statistical analyses are indicated throughout. Specifics of growth rate and survival analysis in GEMM have been previously described [37].

## Results

**Fig 1:**
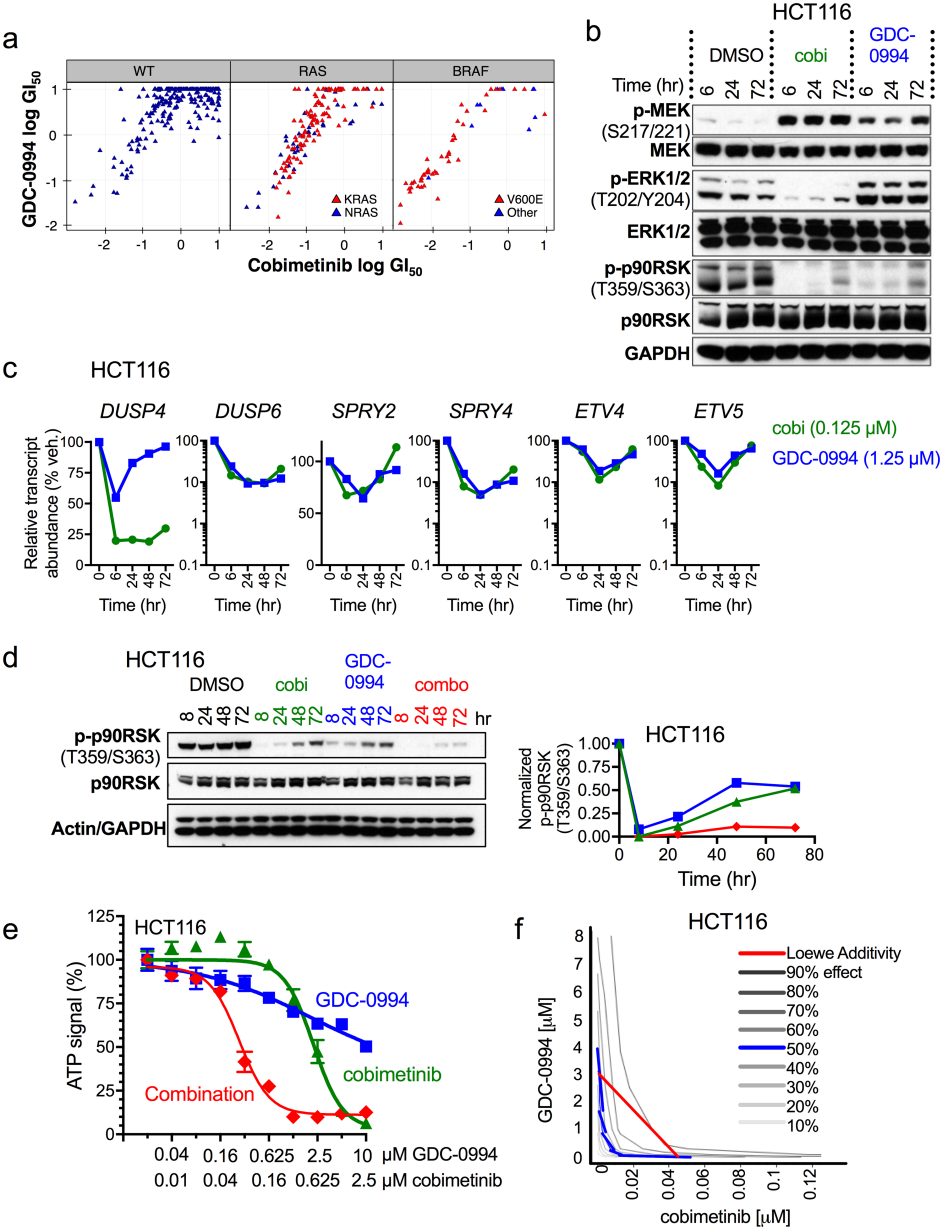
Dual node targeting with ERK and MEK inhibitors prevents MAPK pathway reactivation and has synergistic activity in *KRAS* mutant cells. (**a**) Calculated GDC-0994 and cobimetinib GI_50_ (50% growth inhibition) values across all lines tested are significantly correlated (Pearson correlation R = 0.446, *p* = 1.76 x 10^−24^). Calculated GI_50_ values from large-scale cell viability screening (474 cell lines) using cobimetinib (Y axis) and GDC-0994 (X axis) in cell lines WT for all *RAS* and *RAF* genes, or harboring mutations in any *RAS* or *RAF* genes, respectively. (**b**) Pathway reactivation at the level of p-p90RSK was assessed in *KRAS* mutant cells, HCT116, using single agent cobimetinib at EC_50_ concentrations of 0.25 μM or GDC-0994 at 1.25 μM (HCT116 (*KRAS*^*G13D*^, colorectal)) at indicated time points. **c**, Pathway reactivation at the level of MAPK target gene transcript in HCT116 cells using the same EC_50_ concentrations as in **b** for the indicated time points. (**d**) Pathway reactivation at the level of p90RSK was assessed in *KRAS* mutant cells, HCT116, using single agent cobimetinib 0.25 μM, single agent GDC-0994 1.25 μM or combination treatment using 0.125 μM and 0.625 μM, respectively, at indicated time points. The graph to the right shows quantification of the immunoblot on the left for normalized p-p90RSK ([p-p90RSK/actin]/[total p90RSK/actin]) following treatment with cobimetinib (green triangles), GDC-0994 (blue squares) or the combination (red diamonds) in HCT116 cells. (**e**) HCT116 cells were treated with cobimetinib and GDC-0994 at the indicated concentrations and cell viability was measured after 72 hr of culture (CellTiter-Glo^®^). (**f**) Isobologram analysis of EdU incorporation was utilized to evaluate the combination of cobimetinib and GDC-0994 on HCT116 proliferation. Predicted Loewe additivity is shown in red, whereas fitting of the 50% effect values is plotted in blue.

To investigate the concept of dual node targeting of the MAPK pathway with MEK and ERK inhibitors we assessed the FDA-approved MEK inhibitor, cobimetinib [30], and the ERK inhibitor, GDC-0994, a recently developed potent and selective ATP-competitive inhibitor of ERK1/2 [38] (S1a–S1g Fig). Baseline comparative studies of pathway pharmacodynamics and the impact of ERK (GDC-0994) versus MEK (cobimetinib) inhibition were performed. In a cell viability screen of 474 human cancer cell lines the calculated GI_50_ values for GDC-0994 and cobimetinib were strongly correlated, with *BRAF* mutant cell lines showing the strongest sensitivity and *BRAF/RAS*-wild type (WT) and *RAS* mutant lines showed more variable and modest potency, suggesting redundancy and overlapping pharmacological effects of MEK and ERK inhibitors (Fig 1a). Comparative pathway pharmacodynamics studies illustrated the expected effects with cobimetinib inhibiting MEK and suppressing downstream pERK and p-p90RSK, and GDC-0994 inhibiting ERK and suppressing downstream p-p90RSK (Fig 1b). Similarly, both cobimetinib and GDC-0994 demonstrate transient suppression of multiple MAPK target genes, including *DUSP4*, *DUSP6*, *SPRY2*, *SPRY4*, *ETV4*, and *ETV5*, with levels recovering in a manner consistent with pathway reactivation (Fig 1c). Consistent with previous studies [22,23], pathway reactivation was apparent for MEK inhibitor by 24–72 hrs in *KRAS* mutant cells (Fig 1d and S2c Fig), but not in *BRAF* mutant cells (S2a and S2b Fig). Similarly, ERK inhibition alone also only transiently suppressed the MAPK pathway, demonstrating clear p-p90RSK accumulation by 72hrs (Fig 1b). Pathway rectivation was not due to drug instability and replacement of fresh drug-containing media every 24 hr did not alter the outcome (data not shown). Collectively these data demonstrate that *KRAS* mutant tumor lines respond similarly to MEK or ERK inhibition and that pathway reactivation would be expected to limit the activity of single agent approaches.

Since single node MEK or ERK targeting in *KRAS* mutant setting demonstrates clear pathway reactivation, we next examined effects of combining MEK and ERK inhibition.

The precise definition of synergy with respect to combined actions of two drugs is complex and an ongoing topic of debate (see [39] for a comprehensive discussion). As a general principle however, synergy refers to a fractional effect significantly in excess of the ‘additive’ or ‘independent’ effects of two compounds with independent mechanisms of action. In the case of two agents acting on different nodes in the same pathway, Loewe independence is the preferred null hypothesis. However, estimation of effects in this model requires a robust set of dose-response curves for each single agent and different fraction-dose combinations [40]. For comparing fractional effects of single agents and combined doses at fixed doses, additivity as defined by Bliss, while not strictly correct [40], provides an acceptable approximation of a null hypothesis.

Using the latter criteria, the effects of combined versus single ERK and MEK inhibitor treatment on the most ERK-proximal downstream target, p90RSK, were evaluated. This revealed that combination treatment resulted in more robust and durable pathway suppression than either single agent even when using factional dose concentrations of each drug (i.e. half of that used for single agents) (Fig 1d and S2c Fig).

To explore the functional outcome of these signaling effects, we assessed cell viability relative to a therapeutic treatment dose. In a set of *KRAS* mutant cell lines, the combination of cobimetinib and GDC-0994, compared to the fractional effect of each single agent at each informative point on the dose-response curves was consistent with a greater-than-additive effect. In consequence, there were considerable shifts in GI_50_ (Fig 1e and S3a Fig). Similar combination effects were observed when cobimetinib was combined with other ERK inhibitors, including SCH772984, VX-11e, and ulixertinib (S4a Fig) or when GDC-0994 was combined with other MEK inhibitors, including GDC-0623, selumetinib, trametinib and binimetinib (S4b Fig). Confirmation of synergy in the interaction of cobimetinib and GDC-0994 in inhibiting cell proliferation in multiple *RAS* mutant cell lines was demonstrated by carrying out two-way dose matrix experiments with an EdU-incorporation for DNA synthesis [32] and analyzing the data by the Loewe additivity model [39] [40] (Fig 1f and S3b–S3e Fig). These data suggest that in the *RAS* mutant setting, where pathway reactivation limits MAPK pathway targeting, dual node MEK and ERK inhibition significantly improves MAPK pathway suppression, preventing pathway reactivation and synergistically reducing cell proliferation.

**Fig 2:**
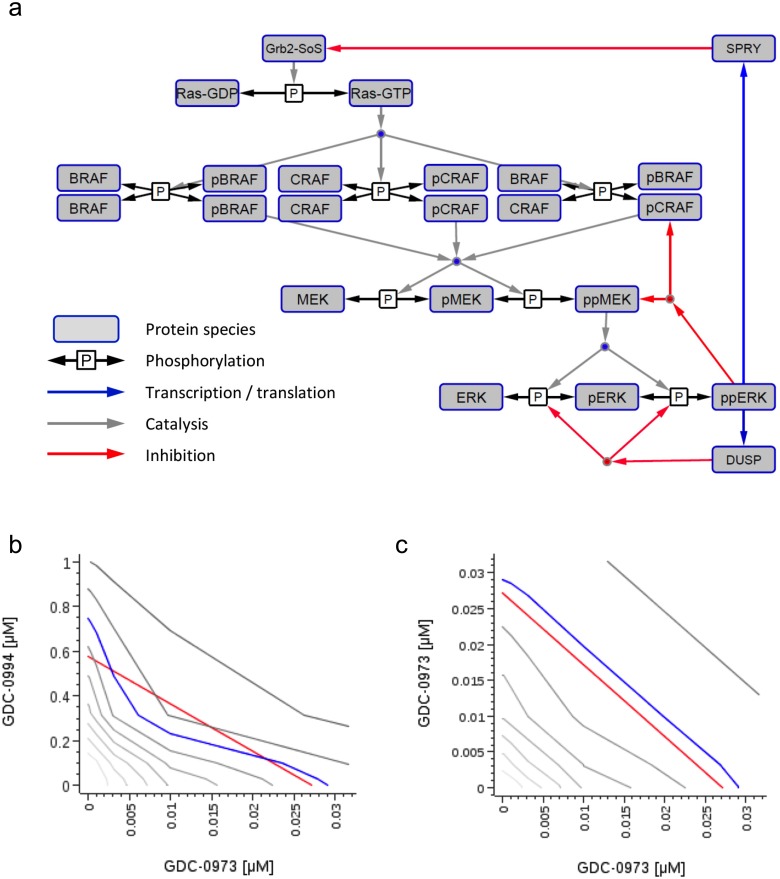
MAPK signaling network model simulations predict synergistic activity between cobimetinib and GDC-0994. (**a**) MAPK signaling model schematic, wherein the system input Grb2-SOS induces catalysis of Ras-GDP to Ras-GTP, which then catalyzes a phosphorylation cascade from BRAF/CRAF dimers via MEK to ERK, with ppERK as the system output. Three canonical negative feedback mechanisms are considered; inhibitory phosphorylation of MEK and CRAF by ppERK, DUSP-mediated ERK de-phosphorylation, and SPRY-mediated inhibition of Grb2-SOS/RAS signaling. (**b**, **c**) Fractional cell viability was predicted in the presence of combined doses of two drugs ranging from 1 to 1000 nM in half-log dilution steps to form a 9x9 matrix; Isobolograms show the predicted effect on cell viability at each dose level (grey lines), with blue line showing 70% effect compared to expected effect for Loewe additivity (red line). Combining GDC-0994 with cobimetinib (**b**) results in deviation from additivity, combining cobimetinib with itself (**c**) demonstrates that an additive effect conforms to the expected Loewe model.

To evaluate the hypothesis that greater than additive effects of dual-node inhibition could be an intrinsic property of the local MAPK signaling network, we developed a mass-action kinetics-based biochemical model of the MAPK cascade connecting the GrB2-SoS signaling node to pp-ERK (Fig 2a and S1 Table), based on prior publications [41] [42]. Experimentally determined binding affinities and reactions for GDC-0994 and cobimetinib were incorporated into the model to simulate in vitro cell viability of a *KRAS* mutant cell line in response to a full combination treatment matrix of cobimetinib and GDC-0994 (Fig 2b). The predicted response, analyzed in reference to the Loewe additivity model [36] demonstrated synergistic activity between the two compounds, as illustrated by isobologram analysis (Fig 2b). The ability of the model to differentiate between additive and synergistic combination effects was demonstrated by predicting the response to a ‘sham’ combination of cobimetinib combined with itself, which as expected [39] resulted in a strictly additive response in which the response surface isoboles are straight lines (Fig 2c). Thus the structural and dynamic properties of the signaling network are sufficient to generate a synergistic sensitivity to combined MEK and ERK inhibition. This implies that the experimentally observed synergy is not attributable to an idiosyncratic feature of the cell lines chosen or the drugs, but it is a property embedded in the RAS-MAPK network.

**Fig 3:**
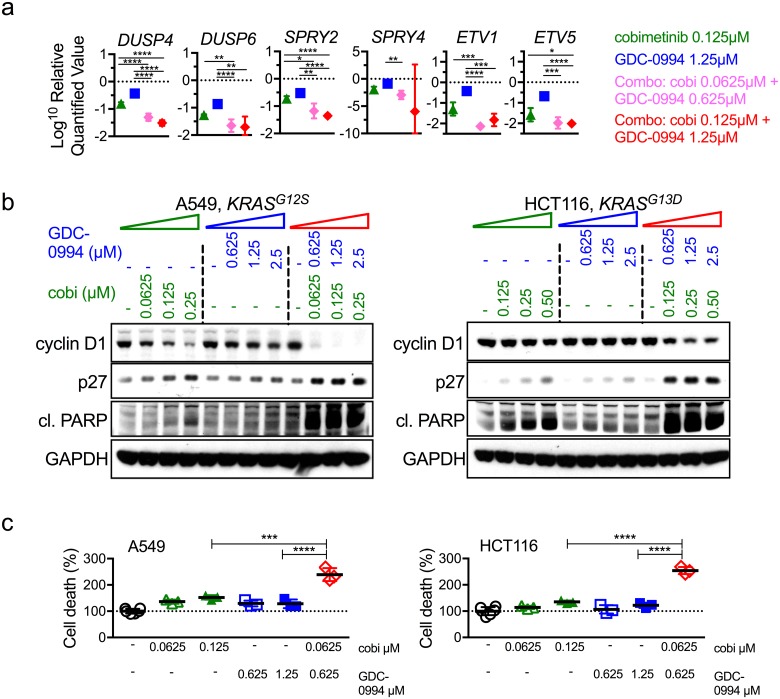
Combination of MEK and ERK inhibitors results in stronger suppression of MAPK pathway output and overcomes pathway reactivation. (**a**) MAPK target genes *DUSP4*, *DUSP6*, *SPRY2*, *SPRY4*, *ETV1*, and *ETV5* expression following 24hr treatment with the indicated doses in A549 cells. One-way ANOVA, * p<0.05, **p<0.01, *** p<0.005, **** p<0.001. (**b**) The combination cobimetinib and GDC-0994 in HCT116 and A549 (*KRAS*^*G12S*^, NSCLC) cells results in stronger reduction of cyclin D1 accumulation, increased p27 levels, increased cleaved PARP levels, and, **c**, increased cell death at 24 hrs post-treatment at the concentrations indicated. One-way ANOVA, *** p<0.005, **** p<0.001.

To further elucidate the mechanistic underpinning of the synergistic behavior observed in the dual node treatment, we assessed treatment impact on transcriptional targets of MAPK pathway. Combining fractional doses (i.e half of that used for single agents) resulted in suppression of MAPK target genes to a greater degree than single-agent treatments (Fig 3a, pink relative to blue or green). Moreover, a time course to 72 hr revealed that the kinetics following combination treatment are significantly delayed and transcripts do not recover to expression levels of baseline (5 out of 7 transcripts recover <4% of their baseline values, *DUSP4* recovers to 7.5% and *SPRY2* recovers 58%), in contrast to single agent treatments where transcript levels recover in step with pathway reactivation (S5 Fig). Together these results demonstrate a more robust target gene expression decrease, as well as significantly delayed recovery observed with combination treatment that cannot be matched by increased dosing of either single agent treatment.

Strikingly, the uniqueness of the combination treatment impact was also observed when assessing direct functional pathways readouts, including cell cycle markers, such as cyclin D1, p27 and PARP cleavage. In all cases, the impact of the lowest fractional combination dose surpassed that of two times higher doses of either single agent. Combination treatment resulted in decreased cyclin D1, increased p27 and increased cleaved PARP (Fig 3b and 3c). In *BRAF* mutant cell lines cobimetinib and GDC-0994 were much more effective as single agents and combination similarly led to suppressed p-p90RSK and cyclin D1 (S6a Fig), however fractional doses failed to induce higher levels of apoptosis, but higher dose intervals of 4x did increase apoptosis (S6b Fig). These data emphasize that dual node targeting is more than additive in its impact on signaling and functional outcome in MAPK dysregulated cancer cells, particularly in the *RAS* mutant setting.

**Fig 4:**
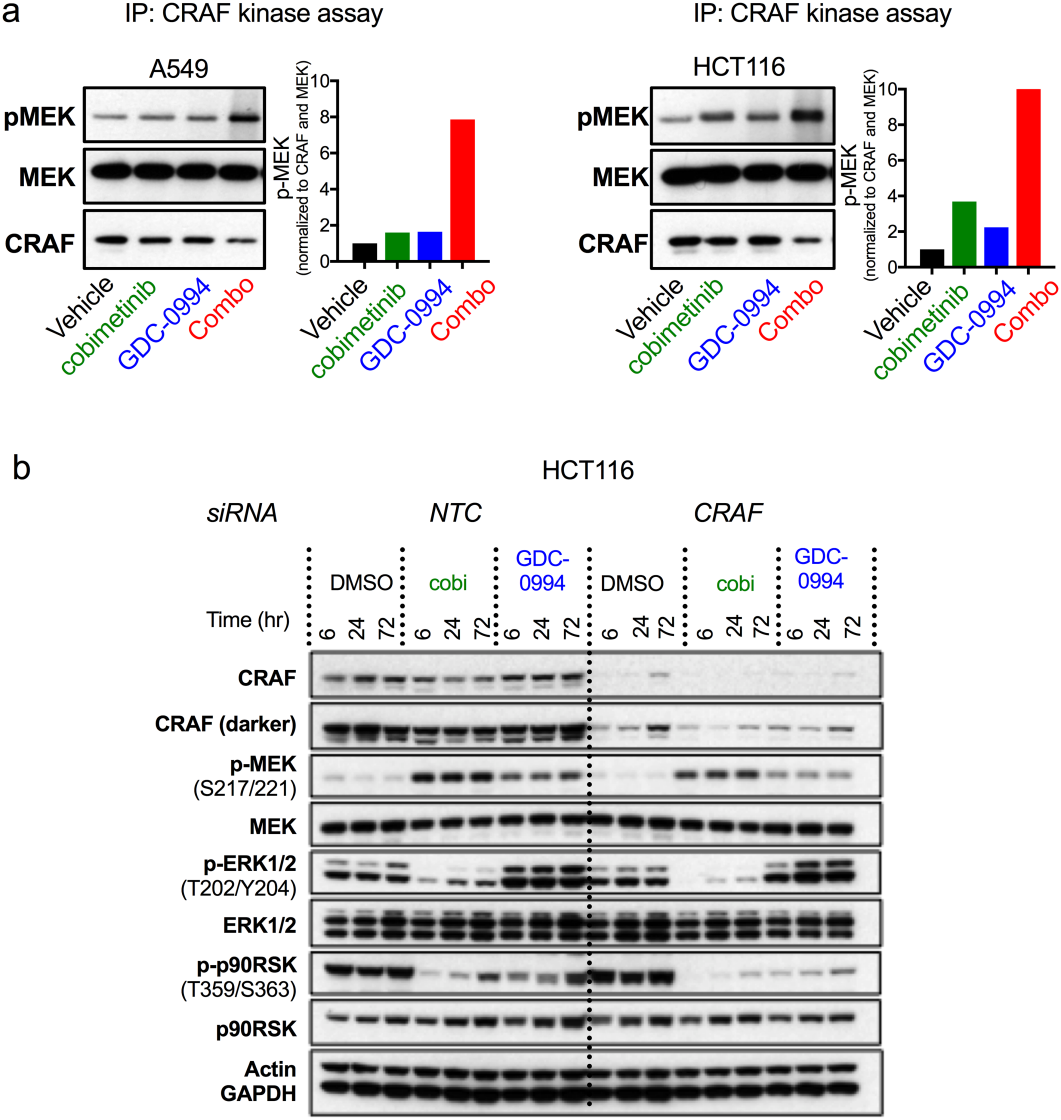
CRAF plays a key role in pathway reactivation following MEK and ERK inhibition. (**a**) CRAF kinase activity measured following single or dual node targeting in A549 (cobimetinib: 0.125μM, GDC-0994: 1.25μM, combination: 0.0625 μM and 0.625 μM, respectively) and HCT116 (cobimetinib: 0.25μM, GDC-0994: 1.25μM, combination 0.125 μM and 0.625 μM, respectively) for 6hr. Quantification of Western data using ImageJ are shown next to the Western blot. This is a representative image from five independent experiments. (**b**) HCT116 cells transfected with either a non-targeting control (NTC) or CRAF siRNA were treated 24 hr post electroporation with cobimetinib (0.25 μM) or GDC-0994 (1.25 μM) for 6, 24, and 72 hr.

In the case of single agent MEK inhibitor treatments, previous work has identified the loss of feedback control of CRAF activity as a critical liability for MAPK pathway feedback reactivation [22] [23]. This prior work concluded that inhibition of CRAF-MEK signaling axis is necessary for sufficient MAPK pathway suppression in the *KRAS* mutant context. Consistent with previous work, cobimetinib treatment increased CRAF kinase activity approximately 2- to 4-fold in *KRAS* mutant cell lines (Fig 4a). Single-agent ERK inhibition with GDC-0994 also increased CRAF kinase activity approximately 2-fold, consistent with our observation that ERK inhibition is similarly susceptible to pathway reactivation. We hypothesized that dual node inhibition would cause greater suppression of negative regulators of upstream MAPK signaling (e.g. SPRY and DUSP expression and CRAF phosphorylation) and this would in turn increase CRAF kinase activity beyond that of either single node treatment. Indeed, combination treatment resulted in an almost 10-fold induction of CRAF kinase activity (Fig 4a). Knockdown of *CRAF* via siRNA demonstrated improved suppression of downstream signaling when combined with either cobimetinib or GDC-0994, consistent with a key role for CRAF in mediating pathway reactivation upon inhibition of either MEK or ERK (Fig 4b). These data also demonstrate that dual node targeting at the level of RAF with either MEK or ERK could also be an effective means of more durably suppressing dysregulated MAPK signaling. Taken together, these findings indicate that despite highly elevated CRAF activity, robust pathway suppression with dual node targeting is sufficient to prevent transmission of this activity to downstream pathway outputs leading to more potent and durable anti-tumor activity, particularly in the *RAS* mutant setting where CRAF plays a more prominent role in pathway reactivation.

**Fig 5:**
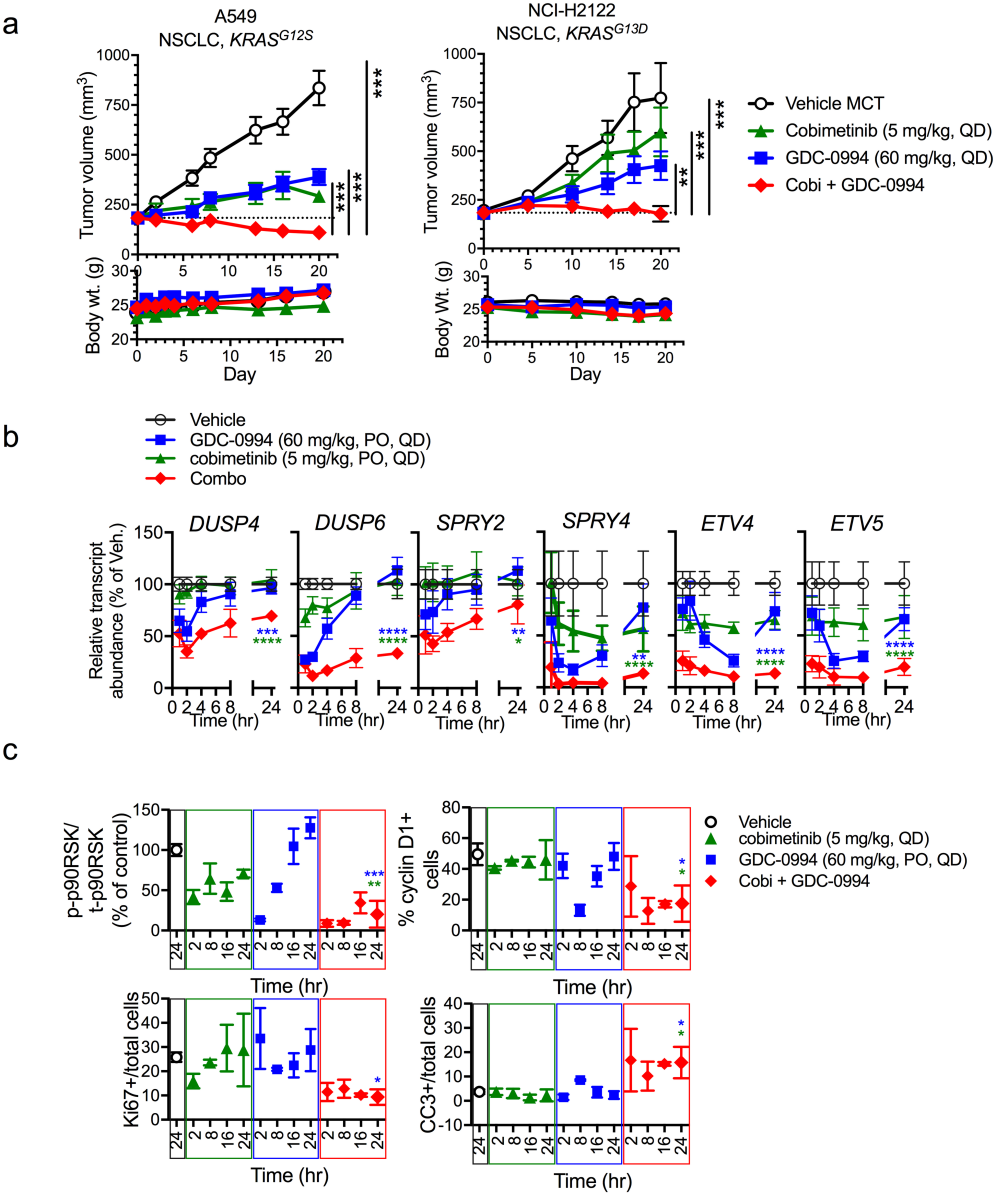
Combination of MEK and ERK inhibitors results in stronger suppression of *KRAS* mutant tumor growth due to improved suppression of MAPK output. (**a**) Combination of cobimetinib and GDC-0994 demonstrates significantly greater anti-tumor activity in multiple *KRAS* mutant tumor models A549 and NCI-H2122 (cobimetinib at 5 mg/kg, PO, QD + GDC-0994 at 60 mg/kg, PO, QD) compared to single agent (upper panels). Mean tumor volume is plotted ± SEM (n = 10 mice per group). Study was terminated on day 20. All treatments were tolerated with minimal body weight loss (lower panels), One way ANOVA, * p<0.05, ** p<0.01, *** p<0.005, ****p<0.001. (**b**) A549 (NSCLC, *KRAS*^*G12S*^) tumor-bearing mice (n = 3 per time point) were treated with GDC-0994 (60 mg/kg, PO, QDx4), cobimetinib (GDC-0994; 5 mg/kg, PO, QDx4) or the combination and then MAPK target genes expression was assessed in tumor samples (Nanostring^®^) and the quantified results are plotted for each individual gene over time. The combination results in deeper, more prolonged suppression of multiple MAPK target genes, including *DUSP4*, *DUSP6*, *SPRY2*, *SPRY4*, *ETV4*, and *ETV5*. Student’s t test at the 24 hr time point, * p<0.05, ** p<0.01, *** p<0.005, ****p<0.001. (**c**) The combination of cobimetinib and GDC-0994 results in stronger and more prolonged suppression of p-p90RSK/total p90RSK phosphorylation (as determined by quantitative western blot), cyclin D1 and Ki-67, as well as increased induction of cleaved caspase 3 (CC3) (as determined by IHC) in A549 xenograft tumors treated for 4 days (values were quantified from n = 4 mice/time point). Student’s t test at the 24 hr time point, * p<0.05, ** p<0.01, *** p<0.005.

To extend our findings, the combination of MEK and ERK inhibitors was evaluated *in vivo* to assess both tolerability and anti-tumor activity. Combination of cobimetinib and GDC-0994 resulted in a significant decrease of tumor growth in multiple *KRAS* mutant xenograft tumor models compared to single agent treatment arms (Fig 5a). Importantly, this combination was well tolerated at these concentrations, however daily combination at the single agent maximum tolerated doses (MTDs) was not (S7 Fig), indicating that dose and schedule may need to be carefully monitored in a clinical setting. Indeed, dosing of cobimetinib twice weekly with daily GDC-0994 resulted in significant combination activity with all doses being well tolerated (S7 Fig). Correlative pharmacokinetics and pharmacodynamics studies in the *KRAS* mutant A549 model demonstrated that the combination resulted in a decrease in transcript levels of multiple MAPK target genes, including *DUSP4*, *DUSP6*, *SPRY2*, *SPRY4*, *ETV4* and *ETV5* (Fig 5b). In agreement with our *in vitro* data, the dual node targeting resulted in stronger and more prolonged suppression of p-p90RSK, reduced cyclin D1 and Ki-67, as well as an increase in cleaved-caspase 3 at 24 hr (Fig 5c and S8 Fig). Similar enhanced anti-tumor combination activity was observed in a *BRAF* mutant (A375.X1) and *NRAS* mutant (IPC-298) melanoma model, suggesting the potential for application of this combination strategy in other MAPK-dysregulated settings (S9 Fig).

**Fig 6:**
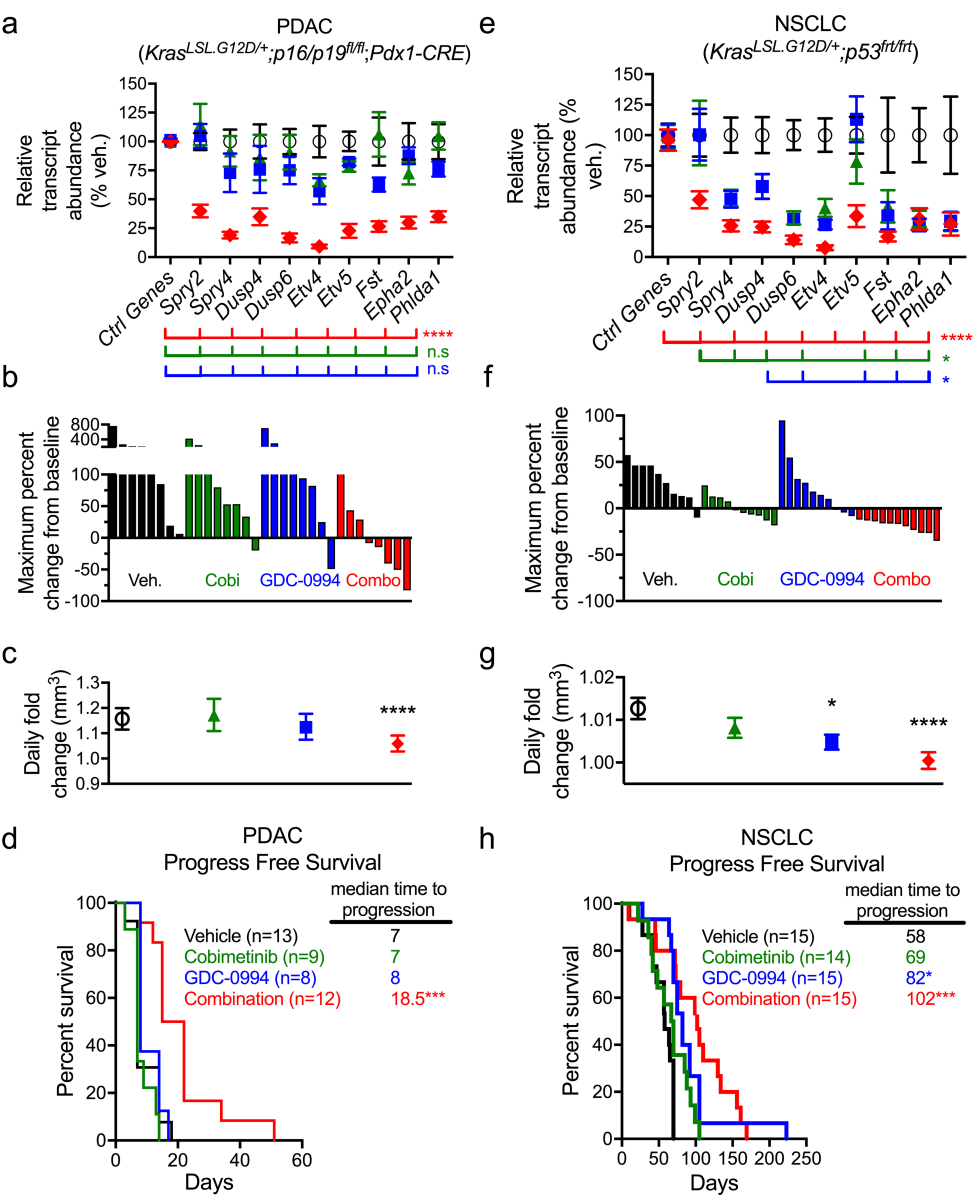
Combination of MEK and ERK inhibitors more potently suppresses *Kras* mutant PDAC and NSCLC GEMM MAPK signaling and tumor progression. In PDAC GEMM, combination of cobimetinib and GDC-0994 (cobi at 5 mg/kg, PO, QD + GDC-0994 at 60 mg/kg, PO, QD) more potently (**a**) reduced MAPK-target gene expression at (6 hr post-last dose following 3 days treatment, n = 4/group); vehicle (black), Cobimetinib (green), GDC-0994 (blue), combination (green); vehicle vs. combo for all genes ****p<0.0001 (red line), n.s., not significant for vehicle vs. GDC-0994 (green line) or Cobi (blue line), Mann-Whitney. (**b)**, Combination treatment improved responses in PDAC model increasing tumor regressions as measured by ultrasound (d7). (**c**) Long-term combo treatment significantly reduced tumor growth rate in PDAC tumors. (vehicle (black) n = 13, Cobimetinib (green) n = 9, GDC-0994 (blue) n = 8, Combination (red) n = 12; ****p = 0.001) (**d**) Long-term combo treatment significantly improved progression free survival in PDAC. Log-rank test, ***p = 0.0023. (**e**) Combination of cobimetinib and GDC-0994 (cobi at 5 mg/kg, PO, QD + GDC-0994 at 60 mg/kg, PO, QD) more potently reduced MAPK target gene expression at (6 hr post-last dose following 3 days treatment, n = 4/group) in NSCLC tumors; vehicle (black), Cobimetinib (green), GDC-0994 (blue), combination (red); vehicle vs. combo. Mann-Whitney, ****p≤0.0001 for all genes (red line); vehicle vs. GDC-0994 *p<0.05 for *SPRY4*, *DUSP6*, *ETV4*, *FST*, *EPHA2*, and *PHLDA1* (green line); vehicle vs. Cobi *p<0.05 for *DUSP6*, *ETV4*, *FST*, *EPHA2*, and *PHLDA1* (blue line); all others n.s., not significant. (**f**) Combination treatment in the NSCLC model increased tumor regressions as measured by micro computed tomography (μCT) (d14) (*p<0.001). (**g**) Long-term combo treatment significantly reduced tumor growth rate in NSCLC tumors (vehicle n = 15, Cobimetinib n = 14, GDC-0994 n = 15, Combination, n = 15; *p<0.05, ****p = 0.0001). (**h**) Reduced tumor growth translated to statistically significant improved progression free survival in NSCLC model (Log-rank test, ***p = 0.004).

We next sought to explore the activity of dual node targeting in *Kras* mutant genetically engineered mouse (GEM) models, including a pancreatic ductal adenocarcinoma (PDAC) model (*LSL-Kras*^*G12D/+*^; *p16/p19*^*fl/fl*^; *Pdx1-CRE*) and a NSCLC model (*LSL-Kras*^*G12D/+*^;*p53*^*frt/frt*^). In the PDAC model, pharmacodynamics analysis revealed a decrease in multiple MAPK target gene transcripts in combination-treated samples, which was not observed for either single agent (Fig 6a). In line with the observed transcriptional impact, neither cobimetinib nor GDC-0994 had a significant effect on the number of tumor responses compared to vehicle (1/8 responses each), consistent with previous reports [36]. In contrast, combination treatment reduced tumor volume in the majority (5/8) of animals (Fig 6b). Analysis of pathway pharmacodynamics in tumor samples demonstrated that only the combination of cobimetinib and GDC-0994 resulted in significant suppression of downstream p-p90RSK (S10 Fig). Consistent with these data, neither cobimetinib nor GDC-0994 treatment significantly reduced long-term tumor growth rate (Fig 6c) or progression-free survival (PFS) (Fig 6d). However combined cobimetinib and GDC-0994 significantly reduced pancreatic tumor growth leading to a significantly improved PFS (7 days in vehicle vs. 18.5 days in combo) (Fig 6c and 6d).

In the NSCLC GEM model, the combination treatment had a similar, but non-overlapping effect on MAPK target gene transcripts compared to the PDAC tumor model, with the combination more deeply suppressing all transcripts, whereas each single agent had a more variable effect (Fig 6e). Cobimetinib and GDC-0994 each resulted in some level of tumor regression (6/10 and 3/10 responses, respectively), whereas the combination resulted in tumor burden reductions in all animals (10/10 tested) (Fig 6f). Pathway pharmacodynamics demonstrated both cobimetinib or GDC-0994 suppressed p90RSK, however the combination resulted in stronger suppression of p90RSK (S10b Fig). Long-term treatment in this model revealed a significant decrease in tumor growth rate in both the combination and GDC-0994 arms (Fig 6g). Consistently, both GDC-0994 and combination treatment improved PFS relative to vehicle control (82 and 102 days vs. 58 days, p<0.05 and <0.0005, respectively) (Fig 6h). Higher variability in the NSCLC model may reflect greater genetic heterogeneity and fundamental differences in pancreatic versus lung tumor development when transformed by mutant *Kras (Chung et al*., *in prep)*. These data demonstrate that the cobimetinib and GDC-0994 dual node combination significantly enhances suppression of MAPK signaling in two *Kras* mutant GEM models, resulting in greater inhibition of tumor growth at well-tolerated doses, ultimately improving animal outcome.

## Discussion

These studies reveal the therapeutic consequences of single node versus dual node targeting in MAPK dysregulated tumors and identify therapeutic strategy of combining MEK and ERK inhibitors which suppress pathway signaling in a deeper and more durable fashion resulting in improve suppression of functional consequences of oncogenic signaling. Analysis of non-oncogenic MAPK signaling has highlighted that multiple feedback mechanisms exist that enables fine-tuning of the signaling cascade and subsequently prevent inappropriate MAPK pathway activity and cell proliferation. When MAPK signaling is chronically activated via oncogenic mutation, cell proliferation becomes largely independent of external mitogenic stimuli, however homeostatic feedback mechanisms remain, including negative regulatory RAF phosphorylation by ERK and expression of Sprouty and DUSPs [9,43–45].

Inhibition of single nodes within the MAPK pathway temporarily attenuates negative feedback, thus increasing the pool of active upstream mediators. In theory, saturating concentrations of a MEK or ERK inhibitor alone would prevent any reactivation via this elevated upstream input. However, in practice, especially *in vivo*, this is likely unattainable due to protein turnover, pharmacokinetics and drug intolerability. Therefore relief of negative feedback can drive pathway activity through the residual fraction of inhibitor-unbound signaling intermediates, ensuring pathway reactivation. Mathematical modeling of this negative-feedback amplifier (NFA)-like pathway illustrates the dynamic nature of the signal transduction networks and further supports the limitations of targeting MEK, the pathway amplifier, which is strongly buffered against perturbations [46]. One observed consequence is that in *RAS*-mutant cells MEK inhibition de-represses CRAF kinase activity through it’s suppression of ERK leading to pathway reactivation over time [22,23]. We have shown here that a similar effect is observed with ERK inhibitors, indicating that this node is not distinct from MEK in this regard, consistent with our modeling data. Similarly, in *BRAF* mutant tumors, other compensatory signaling molecules, such as receptor tyrosine kinases (RTKs), can drive pathway reactivation, leading to persistent MAPK signaling that cannot be permanently and completely repressed by single MAPK node inhibitors [47–50]. The practical consequence of multiple negative and positive dynamic feedback mechanisms in the MAPK pathway is that therapeutic targeting of a single node only partially and transiently suppresses pathway output. Other approaches for mitigating the consequences of MEK inhibitor-induced feedback reactivation in the *KRAS* mutant setting include co-targeting at the level of the receptor, for example in the case of FGFR1 [27,29].

Here we show that dual inhibition of MEK and ERK kinase activity results in a greater than additive effect on downstream biological effects in the *RAS* mutant setting. This is apparent from our data using fractional dose levels to show that dual target inhibition results in more robust suppression MAPK pathway output (p-p90RSK and MAPK target gene expression) than can be achieved with increased single node inhibition. This effect of dual node targeting appears more synergistic in *RAS* mutants than *BRAF* mutant cell lines, consistent with a greater role for CRAF in mediating feedback reactivation in this setting [22,23]. Our findings likely explain the observation of enhanced efficacy with dual MEK ERK inhibition in *NRAS* mutant melanoma cell lines [28]. The above model predicts the combination of CRAF and MEK or ERK inhibitors, would achieve more effective suppression MAPK signaling in the *RAS* mutant setting, as suggested in recent reports [22,23]. Interestingly, with combined MEK and ERK targeting, we observed a striking increase in CRAF activity, beyond that of single node inhibition. Nevertheless, the combination treatment effect is robust enough to prevent subsequent downstream pathway reactivation, indicating that with a sufficient degree of pathway inhibition other nodes may synergize and that CRAF is not unique in this sense. The synergistic effects of dual MEK and ERK inhibition at pharmacologically attainable sub-saturating levels correspond to deeper and more sustained suppression of downstream ERK, consistent with our finding that a therapeutic effect may achievable at lower doses or with intermittent dosing schedules.

While these data are encouraging evidence that dual MEK and ERK inhibition may enable stronger sustained anti-tumor activity in MAPK-addicted tumors, *Kras* mutant GEM tumors do not regress completely and mice do eventually succumb to their tumors despite continuous treatment. Further work will be necessary to understand the innate and acquired mechanisms that determine sensitivity or resistance to dual MEK and ERK inhibition. Another limitation of these findings is the lack of translation of MAPK-related adverse effects in mice. Treatment of patients with MEK inhibitors in the clinic is associated with on-target toxicities of rash, diarrhea and ocular events [51]. It is expected that ERK inhibitors will share these same adverse effects of MEK inhibitors, therefore drawing into question whether combinations of MEK and ERK inhibitors will be tolerated at doses that still achieve a therapeutic benefit. Given the kinetics of target gene suppression in the combination treatment, it is feasible that alternative dosing regiments of combinations of RAF, MEK and/or ERK inhibitors could alleviate the expected overlapping adverse effects. Despite these caveats the combination activity observed here is encouraging and suggests that achieving deeper and more durable suppression of MAPK signaling activity may have substantial benefit at controlling MAPK-dysregulated tumor growth.

## Supporting information

S1 Table Mathematical modeling of the MAPK pathway. (PDF)

S1 Fig ERK inhibitor GDC-0994 molecule summary. (PDF)

S2 Fig Cobimetinib and GDC-0994 activity in *BRAF* mutant cell lines and combination activity in the A549 *KRAS* mutant cell line. (PDF)

S3 Fig Isobologram analysis of dual node targeting in *KRAS* mutant cell lines. (PDF)

S4 Fig Combinations of different MEK and ERK inhibitors results in combination anti-proliferative activity. (PDF)

S5 Fig Dual node targeting leads to deeper target gene suppression and overcome reactivation of transcripts in *KRAS* mutant cell line. (PDF)

S6 Fig Combination of MEK and ERK inhibitors in *BRAF* mutant cell lines. (PDF)

S7 Fig Dose-ranging combination of cobimetinib and GDC-0994 on a daily and intermittent schedule in the HCT116 xenograft tumor model. (PDF)

S8 Fig Immunohistochemistry staining of p-p90RSK, cyclin D1, Ki-67 and cleaved caspase 3 from A549 xenografts treated with cobimetinib and GDC-0994. (PDF)

S9 Fig Combination activity of cobimetinib and GDC-0994 in a *BRAF* and *NRAS* mutant melanoma xenograft model. (PDF)

S10 Fig Combination of cobimetinib and GDC-0994 results in better suppression of p-p90RSK in PDAC and NSCLC GEMM tumors. (PDF)
