# Correction: Combined MEK and ERK inhibition overcomes therapy-mediated pathway reactivation in *RAS* mutant tumors

**DOI:** 10.1371/journal.pone.0192059

**Published:** 2018-01-25

**Authors:** Mark Merchant, John Moffat, Gabriele Schaefer, Jocelyn Chan, Xi Wang, Christine Orr, Jason Cheng, Thomas Hunsaker, Lily Shao, Stephanie J. Wang, Marie-Claire Wagle, Eva Lin, Peter M. Haverty, Sheerin Shahidi-Latham, Hai Ngu, Margaret Solon, Jeffrey Eastham-Anderson, Hartmut Koeppen, Shih-Min A. Huang, Jacob Schwarz, Marcia Belvin, Daniel Kirouac, Melissa R. Junttila

Fig 5a is incorrect. The authors have provided a corrected version here.

**Fig 5:**
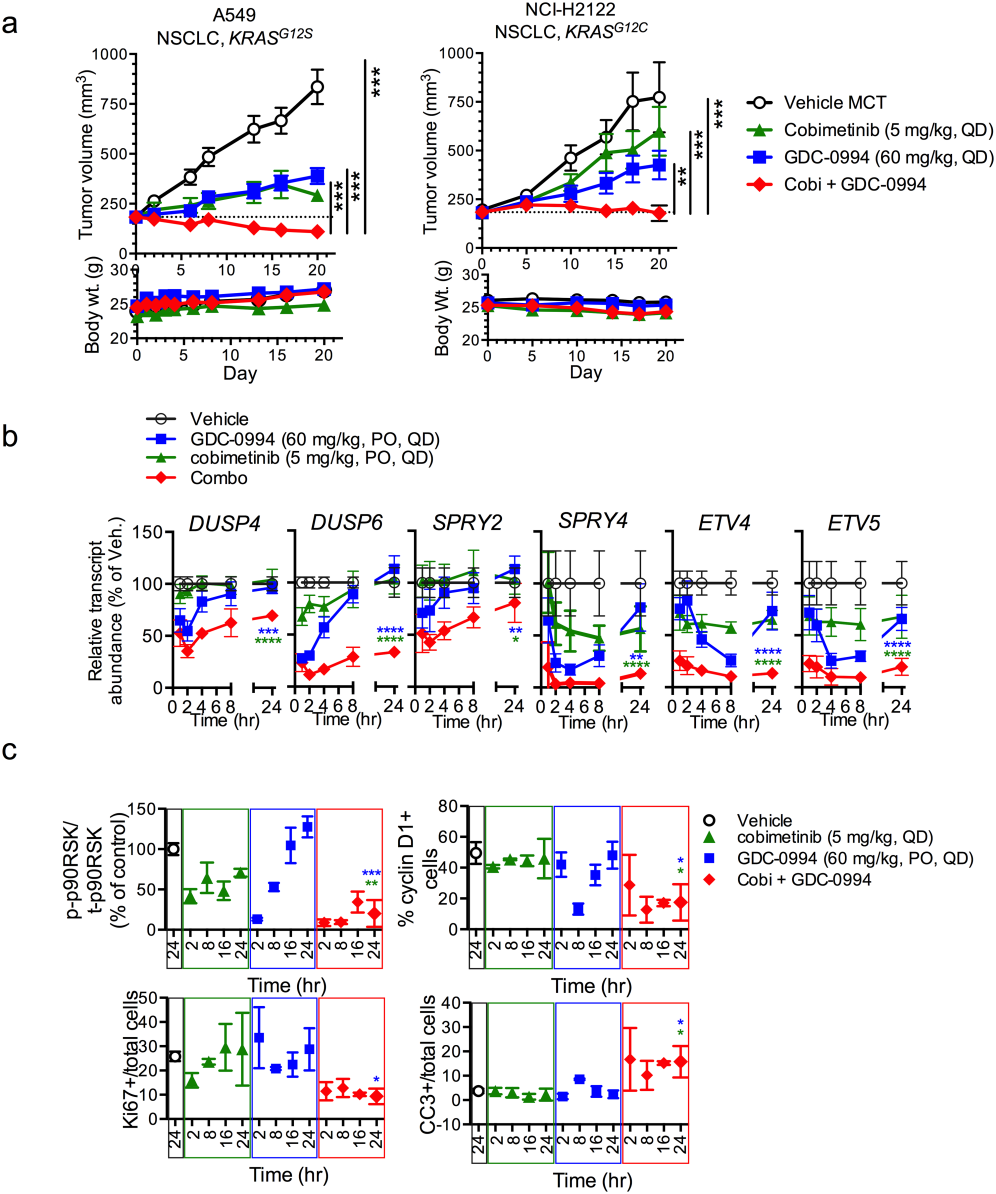
Combination of MEK and ERK inhibitors results in stronger suppression of KRAS mutant tumor growth due to improved suppression of MAPK output. **(a)** Combination of cobimetinib and GDC-0994 demonstrates significantly greater anti-tumor activity in multiple KRAS mutant tumor models A549 and NCI-H2122 (cobimetinib at 5 mg/kg, PO, QD + GDC-0994 at 60 mg/kg, PO, QD) compared to single agent (upper panels). Mean tumor volume is plotted ± SEM (n = 10 mice per group). Study was terminated on day 20. All treatments were tolerated with minimal body weight loss (lower panels), One way ANOVA, * p<0.05, ** p<0.01, *** p<0.005, ****p<0.001. **(b)** A549 (NSCLC, KRAS^G12S^) tumor-bearing mice (n = 3 per time point) were treated with GDC-0994 (60 mg/kg, PO, QDx4), cobimetinib (GDC-0994; 5 mg/kg, PO, QDx4) or the combination and then MAPK target genes expression was assessed in tumor samples (Nanostring^®^) and the quantified results are plotted for each individual gene over time. The combination results in deeper, more prolonged suppression of multiple MAPK target genes, including *DUSP4*, *DUSP6*, *SPRY2*, *SPRY4*, *ETV4*, and *ETV5*. Student’s t test at the 24 hr time point, * p<0.05, ** p<0.01, *** p<0.005, ****p<0.001. **(c)** The combination of cobimetinib and GDC-0994 results in stronger and more prolonged suppression of p-p90RSK/total p90RSK phosphorylation (as determined by quantitative western blot), cyclin D1 and Ki-67, as well as increased induction of cleaved caspase 3 (CC3) (as determined by IHC) in A549 xenograft tumors treated for 4 days (values were quantified from n = 4 mice/time point). Student’s t test at the 24 hr time point, * p<0.05, ** p<0.01, *** p<0.005.

## References

[1] Merchant M, Moffat J, Schaefer G, Chan J, Wang X, Orr C, et al (2017) Combined MEK and ERK inhibition overcomes therapy-mediated pathway reactivation in *RAS* mutant tumors. PLoS ONE 12(10): e0185862 https://doi.org/10.1371/journal.pone.0185862 28982154 10.1371/journal.pone.0185862 PMC5628883

