# Supplementary material for: Combined MEK and ERK inhibition overcomes therapy-mediated pathway reactivation in RAS mutant tumors
Source: PLoS One. 2017 Oct 5;12(10):e0185862. doi: 10.1371/journal.pone.0185862 (PMC5628883; doi:10.1371/journal.pone.0185862)
Supplement: S1 Table — (PDF) [file pone.0185862.s001.pdf]

Mathematical modeling methods

Biochemical reactions and associated parameters related to signal transduction were taken from prior publications ([41] and [42]), and initial concentrations of protein species were taken as median values from quantitative mass spectrometry measurements across a panel of cancer cell lines (Shi et al., 2016). The *KRAS*^{G12V} mutation was implemented via 10-fold reduced binding rate of the active (GTP) form to RAS-GAP. The core model contains 101 reactions and 75 parameters, including the canonical enzymatic cascade (48 parameters) with multiple feedback regulatory loops (27 parameters). An additional 59 reactions related to drug-target interactions were included, and 14 drug-specific parameters were fixed to experimentally measured values. Parameters related to feedback regulatory loops were estimated by fitting *in vitro* phospho-MEK and phospho-ERK dose responses to cobimetinib and GDC-0623 treatments [22] via particle swarm optimization. An empirical relationship was used to transform relative steady state ppERK to cell viability ($1 - e^{-4*(\text{relative ppERK})}$). The model was simulated for 24-hours (to quasi-steady state), followed by drug treatments for 72 hours. The model was coded in SimBiology® and simulated using MATLAB R2016a. Model equations, parameters values, and initial conditions are specified in Supplemental Table 1.

References

Hatzivassiliou, G., J.R. Haling, H. Chen, K. Song, S. Price, R. Heald, J.F.M. Hewitt, M. Zak, A. Peck, C. Orr, M. Merchant, K.P. Hoeflich, J. Chan, S.-M. Luoh, D.J. Anderson, M.J.C. Ludlam, C. Wiesmann, M. Ultsch, L.S. Friedman, S. Malek, and M. Belvin. 2013. Mechanism of MEK inhibition determines efficacy in mutant KRAS- versus BRAF-driven cancers. *Nature*. 501:232–6. doi:10.1038/nature12441.

Schoeberl, B., C. Eichler-Jonsson, E.D. Gilles, and G. Müller. 2002. Computational modeling of the dynamics of the MAP kinase cascade activated by surface and internalized EGF receptors. *Nat. Biotechnol.* 20:370–375. doi:10.1038/nbt0402-370.

Shi, T., M. Niepel, J.E. McDermott, Y. Gao, C.D. Nicora, W.B. Chrisler, L.M. Markillie, V.A. Petyuk, R.D. Smith, K.D. Rodland, P.K. Sorger, W.-J. Qian, and H.S. Wiley. 2016. Conservation of protein abundance patterns reveals the regulatory architecture of the EGFR-MAPK pathway. *Sci. Signal.* 9:1–14.

Sturm, O.E., R. Orton, J. Grindlay, M. Birtwistle, V. Vyshemirsky, D. Gilbert, M.

Calder, A. Pitt, B. Kholodenko, and W. Kolch. 2010. The mammalian MAPK/ERK pathway exhibits properties of a negative feedback amplifier. *Sci. Signal.* 3:ra90. doi:10.1126/scisignal.2001212.

Supplemental Table 1. Mathematical modeling of the MAPK pathway

Parameter	Value	Units
k1f	2.49087E-05	cell/molecule/second
k1r	1.3	1/second
k2	0.5	1/second
k3f	0.0000131	cell/molecule/second
k3r	0.3	1/second
k4	0.023	1/second
k5f	1.6667E-08	cell/molecule/second
k5r	0.0053	1/second
k6f	1.6667E-06	cell/molecule/second
k6r	0.0053	1/second
k7	1	1/second
k8f	0.000118333	cell/molecule/second
k8r	0.2	1/second
k9	1	1/second
k10f	0.000118333	cell/molecule/second
k10r	0.2	1/second
k11	1	1/second
k14	3.5	1/second
k15f	0.00000045	cell/molecule/second
k15r	0.5	1/second
k16	0.058	1/second
k19	2.9	1/second
k20f	2.38333E-05	cell/molecule/second
k20r	0.8	1/second
k21	0.058	1/second
k22f	8.90722E-05	cell/molecule/second
k22r	0.033	1/second
k23	16	1/second
k24f	8.33333E-06	cell/molecule/second
k24r	0.5	1/second
k25	0.246	1/second
k26f	8.90722E-05	cell/molecule/second
k26r	0.033	1/second
k27	5.7	1/second
k28f	0.0000235	cell/molecule/second
k28r	0.6	1/second
k29	0.246	1/second
k30f	0.000000001	cell/molecule/second
k30r	99.20374868	1/second

k31	998.7677413	1/second
k32f	0.003270023	cell/molecule/second
k32r	2.339375737	1/second
k33	29.97830381	1/second
k34f	0.0000099	cell/molecule/second
k34r	1.249296869	1/second
k35	995.6270947	1/second
k36	49.98376987	1/second
k37f	0.000000954	cell/molecule/second
k37r	45.26487127	1/second
k38	20.0666884	1/second
k39f	0.0000005	cell/molecule/second
k39r	11.05212178	1/second
k40	0.005	1/second
k44	0.00001	1/second
k45	0.001	1/second
k46	0.029595942	1/second
k47	0.001072136	1/second
k48f	0.00000211	cell/molecule/second
k48r	65.75095587	1/second
k49f	0.00000244	cell/molecule/second
k49r	49.92687244	1/second
k50	0	1/second
k51f	1.31186E-06	cell/molecule/second
k51r	0.3	1/second
k43	0.002	1/second
k42	0.098260953	1/second
k41	0.005607279	1/second
k401f	3.73E-07	cell/molecule/second
k401r	0.0042	1/second
k402f	4.36E-07	cell/molecule/second
k402r	0.0042	1/second
k13f	7.02424E-07	cell/molecule/second
k13r	0.0162	1/second
k12f	4.0352E-07	cell/molecule/second
k12r	0.0134	1/second
k18f	7.02424E-07	cell/molecule/second
k18r	0.0162	1/second
k17f	4.0352E-07	cell/molecule/second
k17r	0.0134	1/second
k301f	1.66E-06	cell/molecule/second
k301r	5.00E-05	1/second

k302f	1.66E-06	cell/molecule/second
k302r	1.80E-04	1/second
k303f	4.04E-07	cell/molecule/second
k303r	0.67	1/second
k304f	7.02E-07	cell/molecule/second
k304r	0.81	1/second
k305	0.35	1/second
k308f	4.04E-07	cell/molecule/second
k308r	0.67	1/second
k309f	7.02E-07	cell/molecule/second
k309r	0.81	1/second
k310	0.29	1/second
k306f	0	cell/molecule/second
k306r	0	1/second
k307	0	1/second
k311f	0	cell/molecule/second
k311r	0	1/second
k312	0	1/second
