# Supplementary material for: Combined MEK and ERK inhibition overcomes therapy-mediated pathway reactivation in RAS mutant tumors
Source: PLoS One. 2017 Oct 5;12(10):e0185862. doi: 10.1371/journal.pone.0185862 (PMC5628883; doi:10.1371/journal.pone.0185862)
Supplement: S1 Fig — (PDF) [file pone.0185862.s002.pdf]

Figure S1

a

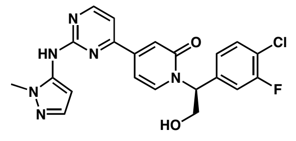

GDC-0994

b

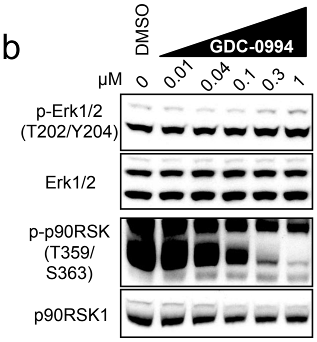

c

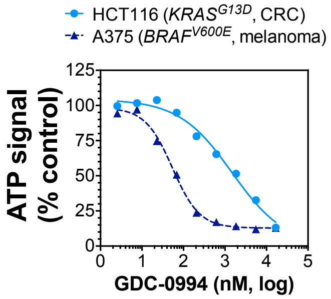

d

Kinase	IC_{50} (μM)
ERK1	0.0012 (Ki)
ERK2	0.003 (Ki)
PrKX	0.061
GSK3α	0.146
CHK2	0.142
HIPK4	0.18
p38δ	0.19
CDK2/Cyclin A	0.20
Aurora A	0.26

e

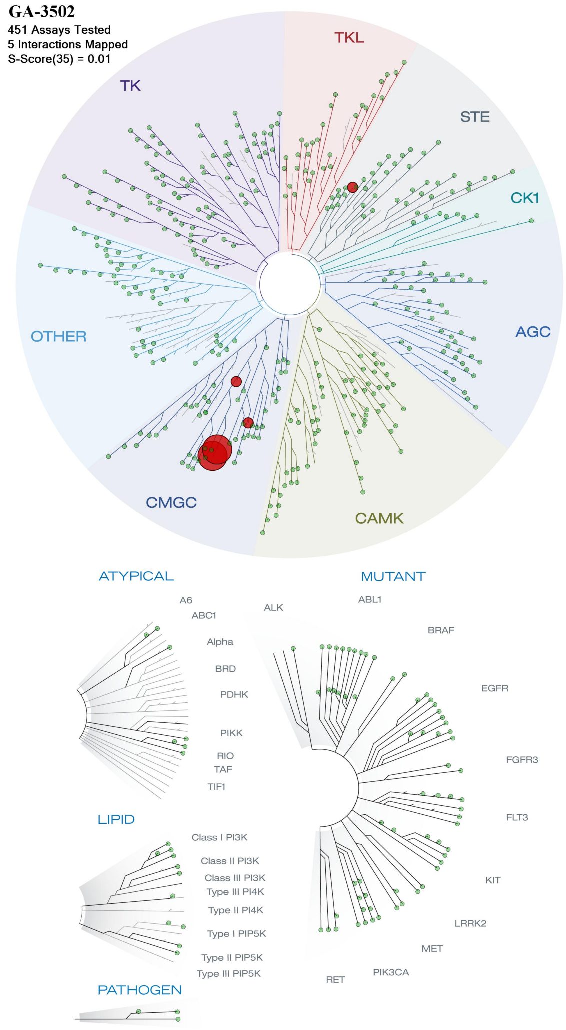

f

Assay	IC_{50} (μM)
P-p90Rsk(Ser380) HepG2	0.012
P-p90Rsk(Ser380) A375	0.07
P-p90Rsk(Ser380) HCT116	0.06
A375 Proliferation	0.083 (GI_{50})
HCT116 Proliferation	3.7 (GI_{50})

g

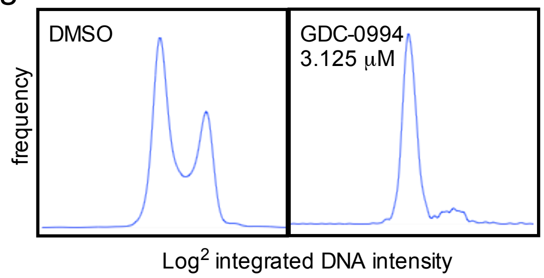

Supplemental Figure 1. ERK inhibitor GDC-0994 molecule summary. (**a**) Chemical structure of ERK1/2 inhibitor, GDC-0994. (**b**) GDC-0994 suppresses

ERK1/2 activity in HCT116 cells, demonstrating suppression of downstream phosphorylation of ERK1/2 substrate, p90RSK while not significantly altering pERK status (treatment 1 hr; concentrations between 0.01 – 1 μM). (**c**) GDC-0994 demonstrates suppression of cell viability in both *RAS* and *BRAF* mutant cell lines in vitro. Exponentially growing cells in medium containing 10% Fetal Bovine Serum (FBS) were treated as indicated for 72 hours, then relative viability was measured using CellTiter-Glo® reagent. (**d**) Enzymatic activity (IC_{50} /Ki values) of GDC-0994 against ERK1 and ERK2 and the next most inhibited kinases are listed. (**e**) Selectivity map of GDC-0994 vs. the kinome as determined using the DiscoverX scanMAX® assay panel (www.discoverx.com). (**f**) GDC-0994 cellular IC_{50} values or inhibition of p90RSK and cellular proliferation in the indicated cell lines. (**g**) GDC-0994 treatment results in a G1 cell cycle arrest in HCT116 cells. Exponentially growing cells in medium containing 10% FBS were treated with 3.125 μM GDC-0994 for 24 hours before paraformaldehyde fixation and staining with Hoechst 33342. DNA content was determined by measuring integrated Hoechst staining intensity for individual cells using Evotec Opera imaging system and Acapella image analysis software.
