# Supplementary material for: Combined MEK and ERK inhibition overcomes therapy-mediated pathway reactivation in RAS mutant tumors
Source: PLoS One. 2017 Oct 5;12(10):e0185862. doi: 10.1371/journal.pone.0185862 (PMC5628883; doi:10.1371/journal.pone.0185862)
Supplement: S2 Fig — (PDF) [file pone.0185862.s003.pdf]

Figure S2

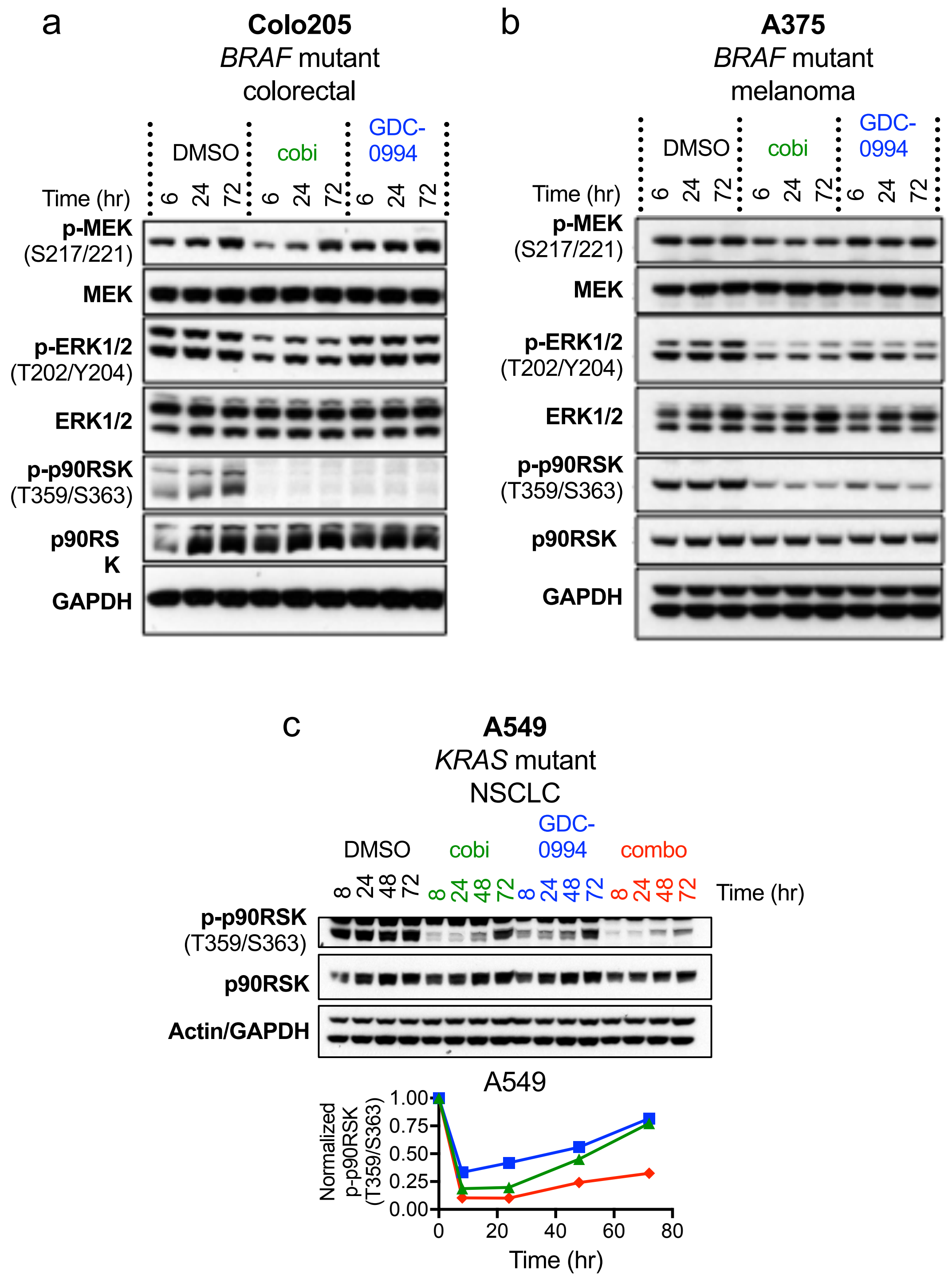

a
Colo205
BRAF mutant
colorectal
DMSO
cobi
GDC-
0994
Time (hr)
6 24 72 6 24 72 6 24 72
p-MEK
(S217/221)
MEK
p-ERK1/2
(T202/Y204)
ERK1/2
p-p90RSK
(T359/S363)
p90RS
K
GAPDH
b
A375
BRAF mutant
melanoma
DMSO
cobi
GDC-
0994
Time (hr)
6 24 72 6 24 72 6 24 72
p-MEK
(S217/221)
MEK
p-ERK1/2
(T202/Y204)
ERK1/2
p-p90RSK
(T359/S363)
p90RSK
GAPDH
c
A549
KRAS mutant
NSCLC
DMSO
cobi
GDC-
0994
combo
8 24 48 72 8 24 48 72 8 24 48 72 8 24 48 72
Time (hr)
p-p90RSK
(T359/S363)
p90RSK
Actin/GAPDH
A549
Normalized
p-p90RSK
(T359/S363)
1.00
0.75
0.50
0.25
0.00
0
20
40
60
80
Time (hr)

Supplemental Figure 2. Cobimetinib and GDC-0994 activity in *BRAF* mutant cell lines and combination activity in the A549 *KRAS* mutant cell line.

(**a**) Colo205 ($BRAF^{V600E}$, colorectal) or (**b**) A375 ($BRAF^{V600E}$, melanoma) cells were treated with DMSO or 0.5x EC50 concentrations of either cobimetinib (0.005 µM) or GDC-0994 (0.1 µM) for the indicated number of hours followed by analysis of pathway activation at the level of MEK, ERK, and p90RSK relative to the loading control GAPDH. (**c**) A549 ($KRAS^{G12S}$, NSCLC) cells were treated with DMSO single agent cobimetinib at 0.25 µM, single agent GDC-0994 at 1.25 µM or the combination of half concentrations of both agents (0.125 µM cobimetinib and 0.625 µM of GDC-0994) for the indicated number of hours followed by analysis of pathway activation at the level of p90RSK relative to the loading controls of Actin and GAPDH. The graph below shows quantification of the immunoblot above for normalized p-p90RSK ([p-p90RSK/actin]/[total p90RSK/actin]) following treatment with cobimetinib (green triangles), GDC-0994 (blue squares) or the combination (red diamonds) in A549 cells.
