# Supplementary material for: Combined MEK and ERK inhibition overcomes therapy-mediated pathway reactivation in RAS mutant tumors
Source: PLoS One. 2017 Oct 5;12(10):e0185862. doi: 10.1371/journal.pone.0185862 (PMC5628883; doi:10.1371/journal.pone.0185862)
Supplement: S3 Fig — (PDF) [file pone.0185862.s004.pdf]

Figure S3

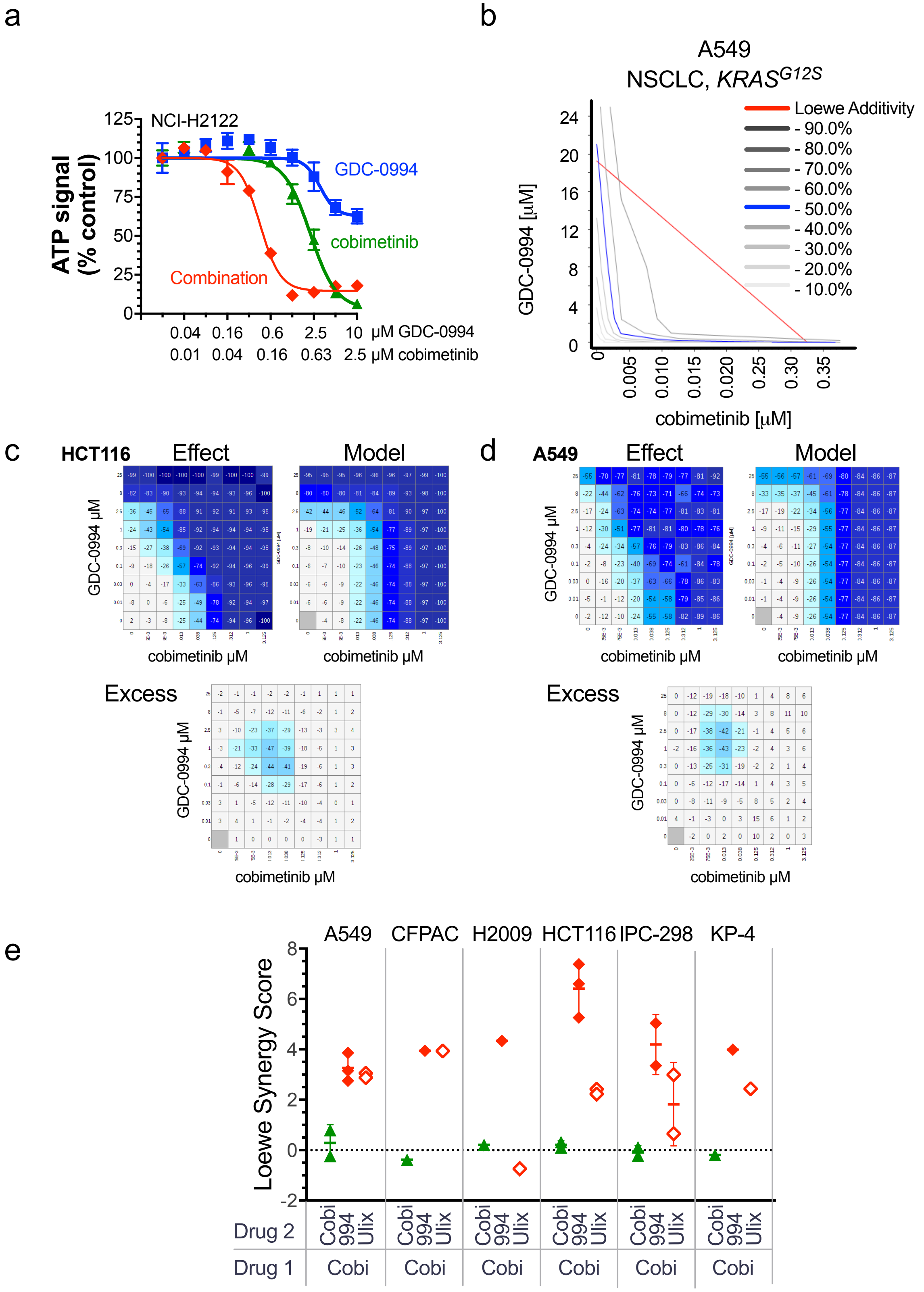

a
b
NCI-H2122
ATP signal (% control)
GDC-0994
cobimetinib
Combination
0.04 0.16 0.6 2.5 10 µM GDC-0994
0.01 0.04 0.16 0.63 2.5 µM cobimetinib
A549
NSCLC, KRAS G12S
Loewe Additivity
- 90.0%
- 80.0%
- 70.0%
- 60.0%
- 50.0%
- 40.0%
- 30.0%
- 20.0%
- 10.0%
GDC-0994 [µM]
cobimetinib [µM]
c
HCT116
Effect
Model
GDC-0994 µM
cobimetinib µM
Excess
d
A549
e
A549 CFPAC H2009 HCT116 IPC-298 KP-4
Loewe Synergy Score
Drug 2 Cobi 994 Ulix
Drug 1 Cobi

Supplemental Figure 3. Dual node targeting is synergistic in *KRAS* mutant setting.

(**a**) Cell viability in NCI-H2122 (*KRAS*^{G12C}, NSCLC) cells were treated with cobimetinib and GDC-0994 at the indicated concentrations and cell viability was measured after 72 hr of culture (CellTiter-Glo®). (**b**) Isobologram analysis of EdU incorporation was utilized to evaluate the combination of cobimetinib and GDC-0994 in A549 (*KRAS*^{G12S}, NSCLC) cells. Predicted Loewe additivity is shown in red, whereas fitting of the 50% effect values is plotted in blue. (**c**) Response data from the isobologram studies in HCT116 (*KRAS*^{G13D}, colorectal) and (**d**) A549 with cobimetinib and GDC-0994 were used to plot the “Effect” vs. the assumed “Model” of additivity with the calculated “Excess” plotted below. (**e**) Synergistic antiproliferative effect of ERK inhibitors with cobimetinib on *RAS* mutant cell lines. Cells were treated with the indicated drug combinations where cobimetinib (Drug 1) was combined with either itself (Cobi, closed green triangles) or the ERK inhibitors GDC-0994 (994, filled red diamonds) or ulixertinib (BVD-523, Ulix, open red diamonds) (Drug 2) in a dose-matrix format. Cells were grown for 48 hours and the proliferative fraction was determined by EdU labeling using high-content imaging. Synergy was determined using the Loewe additivity model and summarized as a weighted average of excess activity over predicted additivity. Cobimetinib combined with itself in both arms of the matrix serves as sham control resulting in a score of zero. Individual symbols represent independent experiments with mean and SD indicated. Cell lines tested: A549 (NSCLC, *KRAS*^{G12C}), CFPAC (pancreatic adenocarcinoma, *KRAS*^{G12V}), H2009 (lung adenocarcinoma *KRAS*^{G12A}), HCT116 (colon carcinoma, *KRAS*^{G13D}), IPC-298 (melanoma, *NRAS*^{Q61L}), KP-4 (pancreatic carcinoma, *KRAS*^{G12D}).
