# Supplementary material for: Combined MEK and ERK inhibition overcomes therapy-mediated pathway reactivation in RAS mutant tumors
Source: PLoS One. 2017 Oct 5;12(10):e0185862. doi: 10.1371/journal.pone.0185862 (PMC5628883; doi:10.1371/journal.pone.0185862)
Supplement: S4 Fig — (PDF) [file pone.0185862.s005.pdf]

Figure S4

a

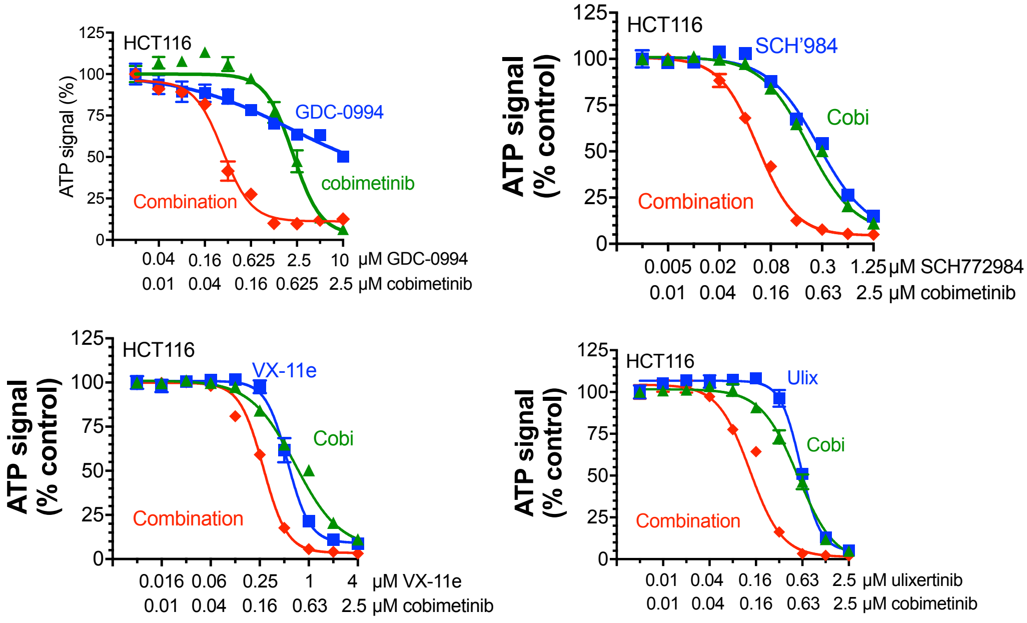

HCT116
GDC-0994
cobimetinib
Combination
ATP signal (%)
0.04 0.16 0.625 2.5 10 µM GDC-0994
0.01 0.04 0.16 0.625 2.5 µM cobimetinib
HCT116
SCH'984
Cobi
Combination
ATP signal (% control)
0.005 0.02 0.08 0.3 1.25 µM SCH772984
0.01 0.04 0.16 0.63 2.5 µM cobimetinib
HCT116
VX-11e
Cobi
Combination
ATP signal (% control)
0.016 0.06 0.25 1 4 µM VX-11e
0.01 0.04 0.16 0.63 2.5 µM cobimetinib
HCT116
Ulix
Cobi
Combination
ATP signal (% control)
0.01 0.04 0.16 0.63 2.5 µM ulixertinib
0.01 0.04 0.16 0.63 2.5 µM cobimetinib

b

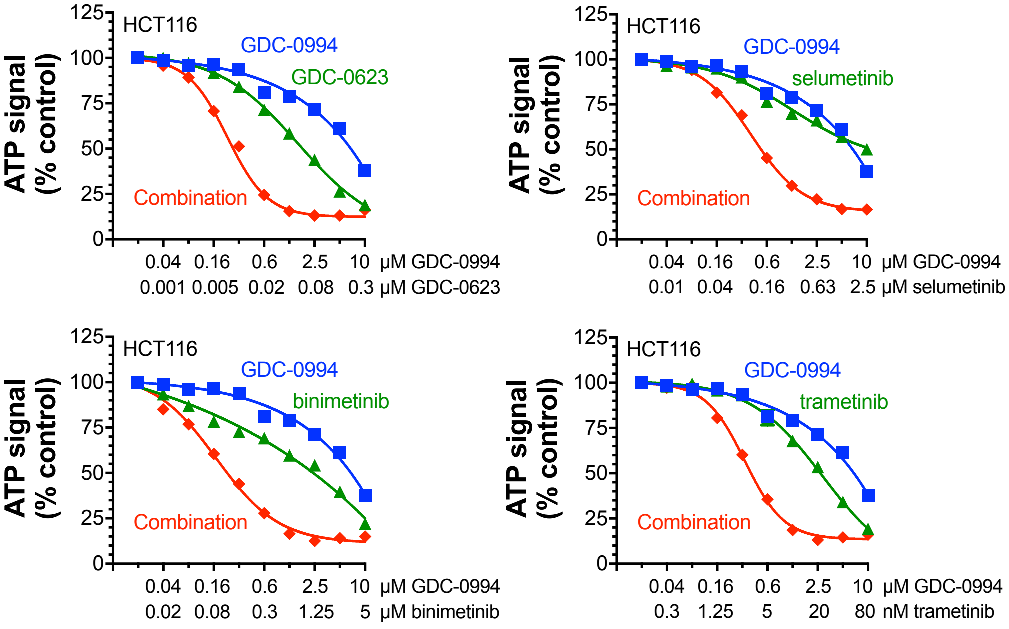

HCT116
GDC-0994
GDC-0623
Combination
ATP signal (% control)
0.04 0.16 0.6 2.5 10 µM GDC-0994
0.001 0.005 0.02 0.08 0.3 µM GDC-0623
HCT116
GDC-0994
selumetinib
Combination
ATP signal (% control)
0.04 0.16 0.6 2.5 10 µM GDC-0994
0.01 0.04 0.16 0.63 2.5 µM selumetinib
HCT116
GDC-0994
binimetinib
Combination
ATP signal (% control)
0.04 0.16 0.6 2.5 10 µM GDC-0994
0.02 0.08 0.3 1.25 5 µM binimetinib
HCT116
GDC-0994
trametinib
Combination
ATP signal (% control)
0.04 0.16 0.6 2.5 10 µM GDC-0994
0.3 1.25 5 20 80 nM trametinib

Supplemental Figure 4. Combination of different MEK and ERK inhibitors results in combination anti-proliferative activity.

(**a**) Cell viability in HCT116 ($KRAS^{G13D}$, colorectal) cells were treated with cobimetinib and four different ERK inhibitors, GDC-0994, SCH772984, Vx-11e, and ulixertinib (BVD-523) at the indicated concentrations and cell viability was measured after 72 hr of culture (CellTiter-Glo®). (**b**) Cell viability in HCT116 ($KRAS^{G13D}$, colorectal) cells were treated with GDC-0994 and four different MEK inhibitors, GDC-0623, selumetinib, binimetinib, and trametinib at the indicated concentrations and cell viability was measured after 72 hr of culture (CellTiter-Glo®). Studies were run concurrently with some curves represented more than once for ease of comparison.
