# Supplementary material for: Combined MEK and ERK inhibition overcomes therapy-mediated pathway reactivation in RAS mutant tumors
Source: PLoS One. 2017 Oct 5;12(10):e0185862. doi: 10.1371/journal.pone.0185862 (PMC5628883; doi:10.1371/journal.pone.0185862)
Supplement: S5 Fig — (PDF) [file pone.0185862.s006.pdf]

Figure S5

a

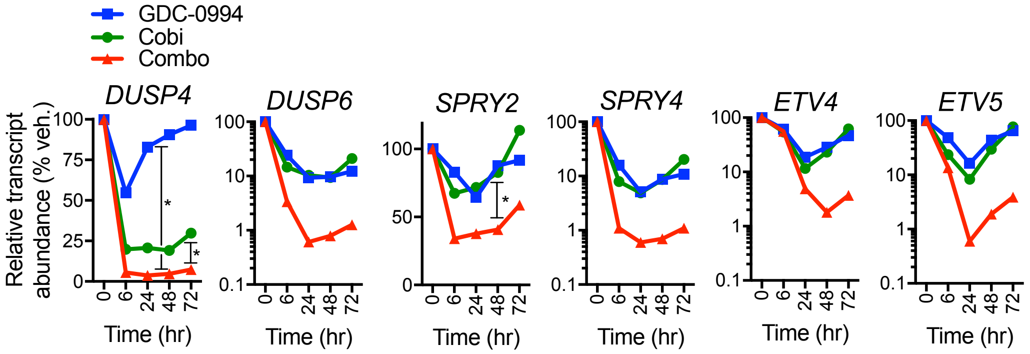

Supplemental Figure 5. Dual node targeting leads to deeper target gene suppression and overcome reactivation of transcripts in *KRAS* mutant cell line. Pathway reactivation at the level of MAPK target gene transcript in HCT116 cells using the EC_{50} concentrations for single agents cobimetinib 0.25 µM, GDC-0994 1.25 µM compared to fractional dual node targeting with cobimetinib 0.125 µM and GDC-0994 0.625 µM at indicated time points. One-way ANOVA, * $p<0.05$.
