# Supplementary material for: Combined MEK and ERK inhibition overcomes therapy-mediated pathway reactivation in RAS mutant tumors
Source: PLoS One. 2017 Oct 5;12(10):e0185862. doi: 10.1371/journal.pone.0185862 (PMC5628883; doi:10.1371/journal.pone.0185862)
Supplement: S6 Fig — (PDF) [file pone.0185862.s007.pdf]

Figure S6

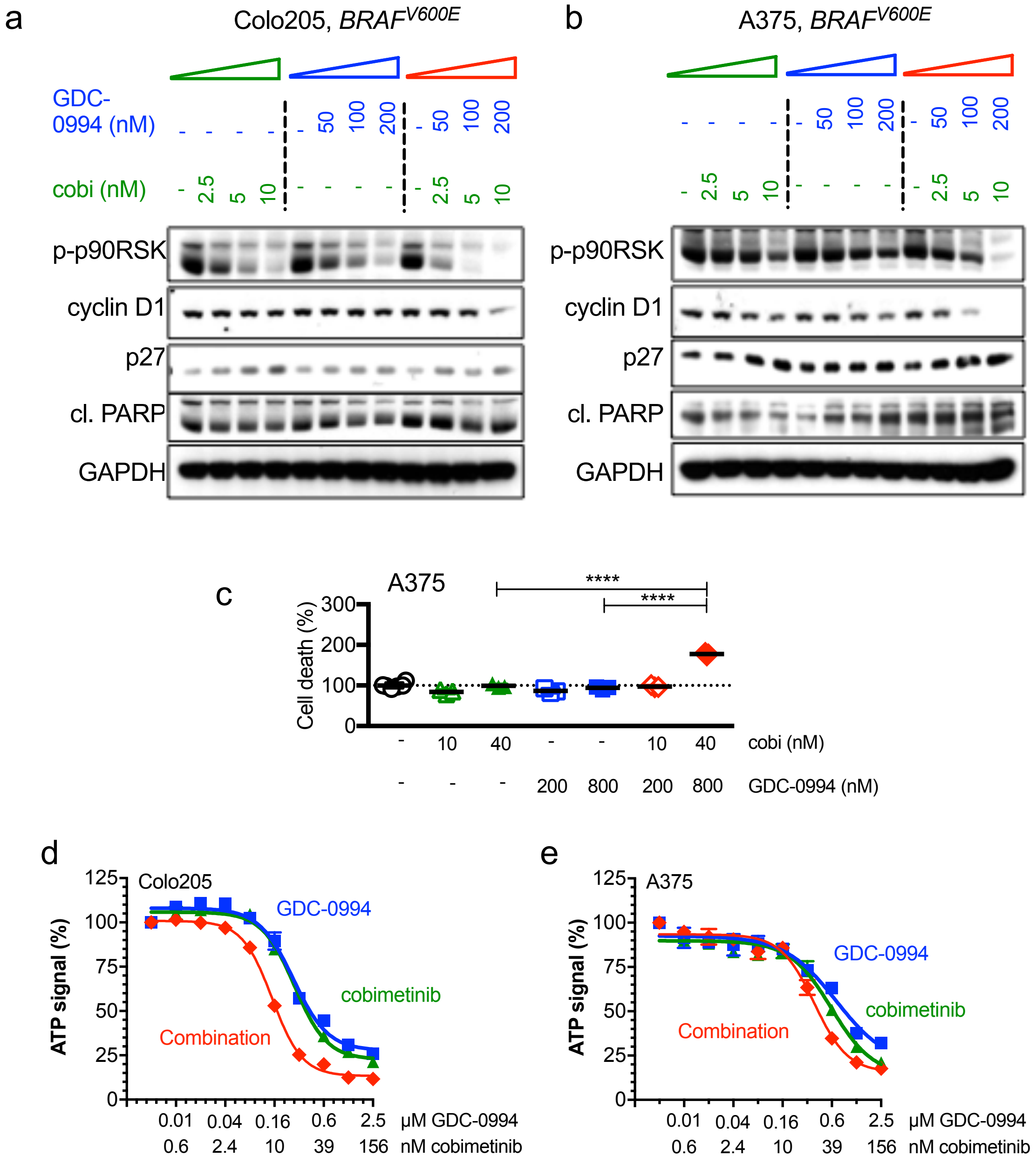
a
Colo205, $BRAF^{V600E}$
GDC-0994 (nM)
cobi (nM)
p-p90RSK
cyclin D1
p27
cl. PARP
GAPDH
b
A375, $BRAF^{V600E}$
c
A375
Cell death (%)
cobi (nM)
GDC-0994 (nM)
d
Colo205
ATP signal (%)
GDC-0994
cobimetinib
Combination
μM GDC-0994
nM cobimetinib
e
A375

Supplemental Figure 6. Combination of MEK and ERK inhibitors in *BRAF* mutant cell lines. (**a**) Colo205 (*$BRAF^{V600E}$*, colorectal) or (**b**) A375 (*$BRAF^{V600E}$*, melanoma) cells were treated with DMSO, cobimetinib, GDC-0994 or the combination at the indicated concentrations for 24 hr followed by analysis of pathway activation at the level of p90RSK, cyclin D1, p27, and cleaved PARP relative to the loading control GAPDH. (**c**) Cell death was measured after 48 hr post treatment (NucELISA) at the indicated concentrations. One-way ANOVA, **** $p<0.001$. (**d**) Colo205 or (**e**) A375 cells were treated with single agent cobimetinib, GDC-0994 or the combination at the indicated fixed ratio concentrations and cell viability was measured after 72 hr in culture (CellTiter-Glo®).
