# Supplementary material for: Combined MEK and ERK inhibition overcomes therapy-mediated pathway reactivation in RAS mutant tumors
Source: PLoS One. 2017 Oct 5;12(10):e0185862. doi: 10.1371/journal.pone.0185862 (PMC5628883; doi:10.1371/journal.pone.0185862)
Supplement: S7 Fig — (PDF) [file pone.0185862.s008.pdf]

Figure S7

a

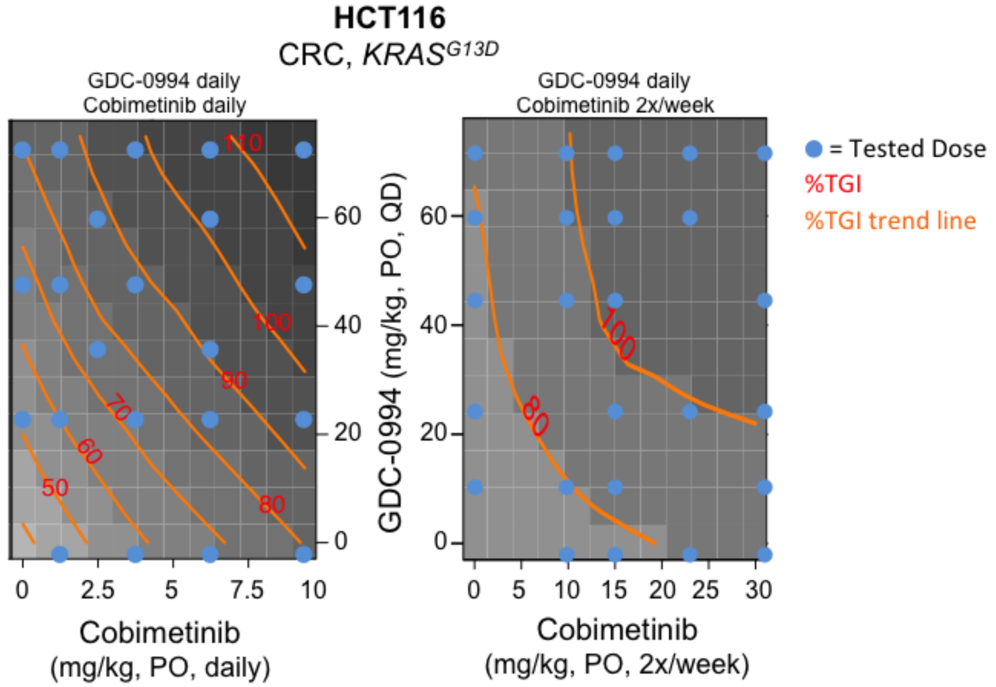

b

%TGI		GDC-0994 (mg/kg)					
		0	25	37.5	50	62.5	75
GDC-0973 (mg/kg)	0	0	38		79		66
	1	48	57		73		74
	2			81		93	
	3	41	73		68		97*
	5	62	85	80		98*	**112***
	7.5	72	100*		**111***		**117***

* p<0.05
Bold red text >15% BW loss

%TGI		GDC-0994 (mg/kg)					
		0	15	30	45	60	75
GDC-0973 (mg/kg)	0	0	84	65	75	82	78
	7.5	44	79		102*		97
	15	78	91	109*	107*	112*	117*
	22.5	75		111*		118*	
	30	93	112		120*		129*

* p<0.05
All combinations were tolerated

Supplemental Figure 7. Cobimetinib and GDC-0994 demonstrate dose-dependent combination activity when dosed on a daily or intermittent schedule in the HCT116 xenograft tumor model. (**a**) HCT116 (*KRAS*^{G13D}, colorectal) tumor xenograft bearing animals were dosed orally (PO) for 21 days with cobimetinib on a daily basis (QD, left panel) or a twice weekly schedule (BIW, right panel) in combination with GDC-0994 PO, QD at the indicated dose levels (blue circles, n=5/group) and %TGI was calculated relative to a vehicle control (n=10). Data were used to generate two-drug contour plots where observed %TGI was used to inform missing dose combinations (%TGI levels indicated in red and %TGI trend lines shown in yellow). Inward curved trend lines indicate greater than additive combination activity. (b) Summary data for the two-drug contour studies depicted above with measured %TGI at each dose combination. Dose combinations resulting in improved %TGI relative to each single agent are indicated (Student's t test, * $p<0.05$). Dose combinations where combinations were considered intolerable are indicated in bold red.
