# Supplementary material for: Combined MEK and ERK inhibition overcomes therapy-mediated pathway reactivation in RAS mutant tumors
Source: PLoS One. 2017 Oct 5;12(10):e0185862. doi: 10.1371/journal.pone.0185862 (PMC5628883; doi:10.1371/journal.pone.0185862)
Supplement: S8 Fig — (PDF) [file pone.0185862.s009.pdf]

Figure S8

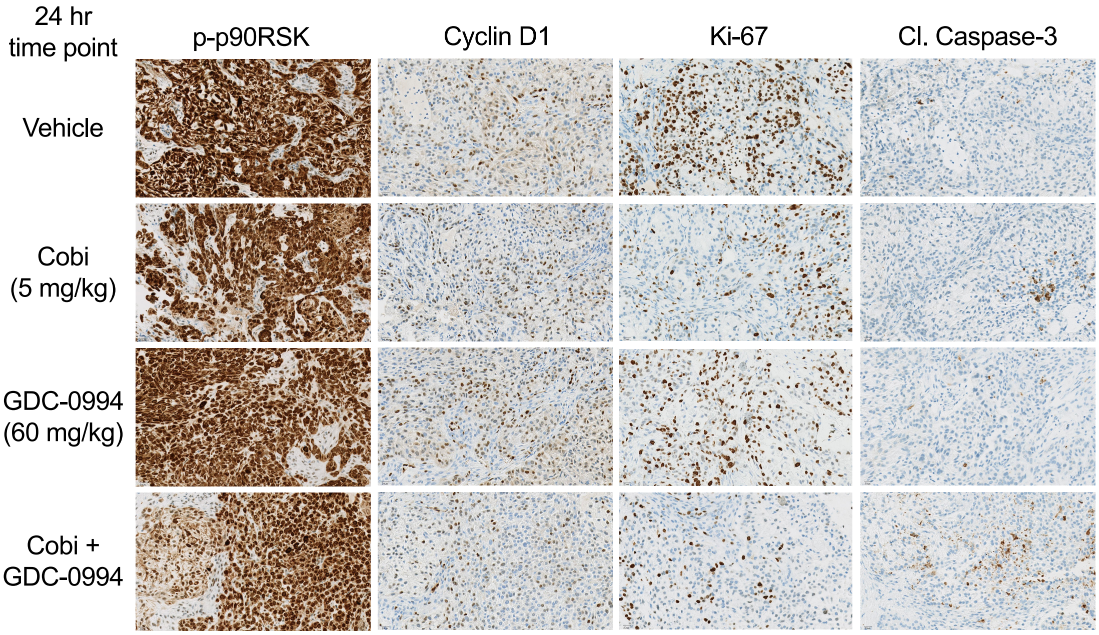

Supplemental Figure 8. Immunohistochemistry (IHC) staining of p-p90RSK, cyclin D1, Ki-67 and cleaved caspase 3 from A549 xenografts treated with cobimetinib and GDC-0994. A549 (*KRAS*^{G12S}, NSCLC) tumor xenograft bearing animals were dosed for 4 days with vehicle, cobimetinib (5 mg/kg, PO, QD), GDC-0994 (60 mg/kg, PO, QD) or the combination (n=4/time point) and IHC for cyclin D1, Ki-67 and cleaved caspase 3 was performed. Slides were scanned and signal was quantified. The figure shows an example of the staining obtained at the 24 hr time point.
