# Supplementary material for: Combined MEK and ERK inhibition overcomes therapy-mediated pathway reactivation in RAS mutant tumors
Source: PLoS One. 2017 Oct 5;12(10):e0185862. doi: 10.1371/journal.pone.0185862 (PMC5628883; doi:10.1371/journal.pone.0185862)
Supplement: S9 Fig — (PDF) [file pone.0185862.s010.pdf]

Figure S9

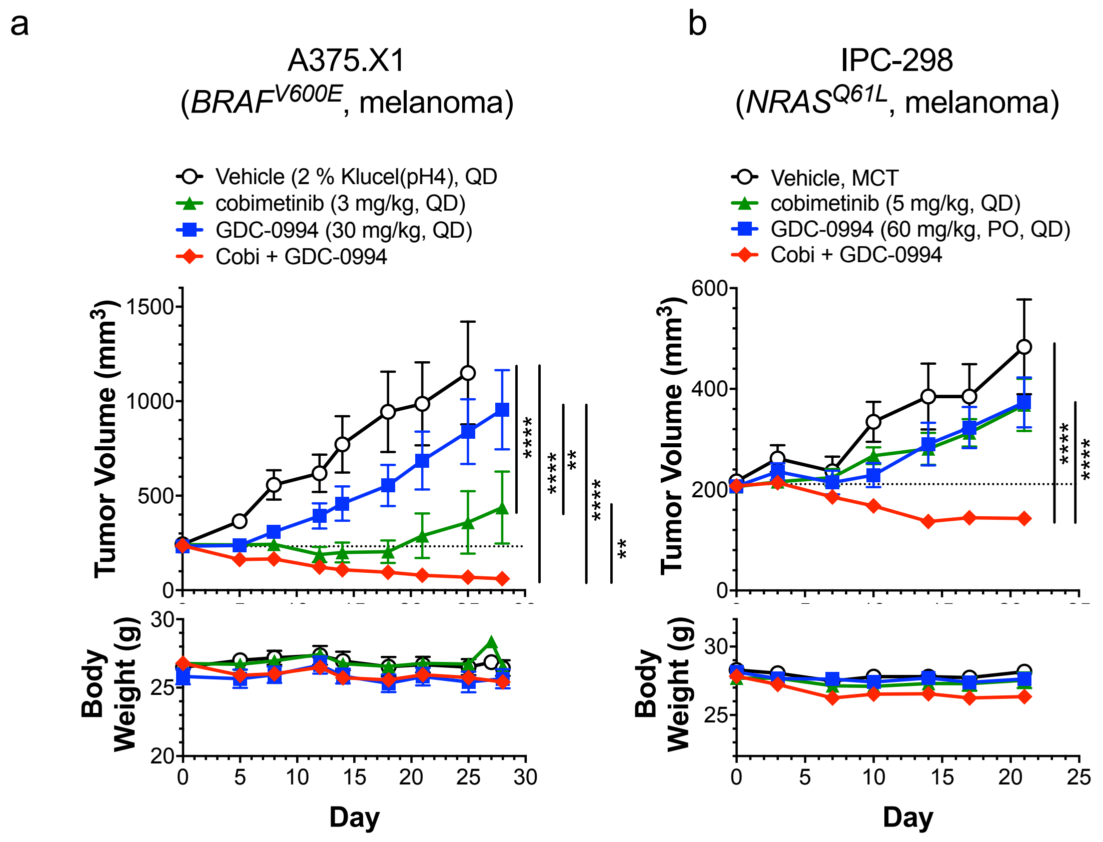

Supplemental Figure 9. Combination activity of cobimetinib and GDC-0994 in a *BRAF* and *NRAS* mutant melanoma xenograft model. (**a**) A375.X1 (*BRAF*^{V600E}, melanoma) tumor xenograft bearing animals (n=10/group) were treated with vehicle, cobimetinib (3 mg/kg, PO, QD), GDC-0994 (30 mg/kg, PO, QD) or the combination for 21 days and tumor growth and body weights were measured. Group mean tumor volume and body weights are plotted ± SEM. (**b**) IPC-298 (*NRAS*^{Q61L}, melanoma) tumor xenograft bearing animals (n=10/group) were treated with vehicle, cobimetinib (5 mg/kg, PO, QD), GDC-0994 (60 mg/kg, PO, QD) or the combination for 21 days and tumor growth and body weights were measured. Group mean tumor volume and body weights are plotted ± SEM. Groups achieving statistical significance are shown (Students t-test, ** p<0.005, *** p<0.001, **** p<0.0001).
