# Supplementary material for: Combined MEK and ERK inhibition overcomes therapy-mediated pathway reactivation in RAS mutant tumors
Source: PLoS One. 2017 Oct 5;12(10):e0185862. doi: 10.1371/journal.pone.0185862 (PMC5628883; doi:10.1371/journal.pone.0185862)
Supplement: S10 Fig — (PDF) [file pone.0185862.s011.pdf]

Figure S10

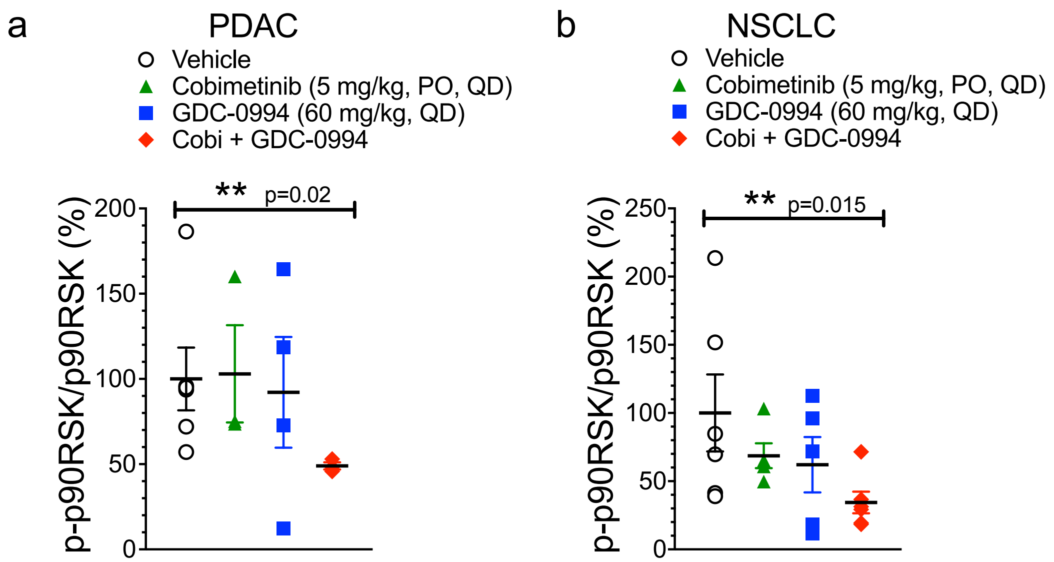

Supplemental Figure 10. Combination of cobimetinib and GDC-0994 results in better suppression of p-p90RSK in PDAC and NSCLC GEMM tumors. (**a**) Quantitative western blot analysis was performed and values for p-p90RSK/total p90RSK (as percent) are plotted from vehicle, cobimetinib, GDC-0994 and the combination of cobimetinib and GDC-0994 treated PDAC tumors (6 hr post-last dose following 3 days of treatment, n=3-5/group, as indicated). One-way ANOVA, p=0.002. (**b**) Similar pathway pharmacodynamics analysis was performed in lung tumor samples from the NSCLC GEMM model (6 hr post-last dose following 3 days of treatment, n=3-5/group, as indicated). In the NSCLC samples, greater pathway suppression was observed with single agent treatments. One-way ANOVA, p=0.002.
